# Family Life in Lockdown

**DOI:** 10.3389/fpsyg.2021.687570

**Published:** 2021-08-04

**Authors:** Pietro Biroli, Steven Bosworth, Marina Della Giusta, Amalia Di Girolamo, Sylvia Jaworska, Jeremy Vollen

**Affiliations:** ^1^Department of Economics, University of Zurich, Zurich, Switzerland; ^2^Department of Economics, University of Reading, Reading, United Kingdom; ^3^Department of Economics, University of Birmingham, Birmingham, United Kingdom; ^4^Department of English Language and Applied Linguistics, University of Reading, Reading, United Kingdom

**Keywords:** lockdown, care, housework, tensions, COVID-19

## Abstract

The lockdown imposed following the COVID-19 pandemic of spring 2020 dramatically changed the daily lives and routines of millions of people worldwide. We analyze how such changes contributed to patterns of activity within the household using a novel survey of Italian, British, and American families in lockdown. A high percentage report disruptions in the patterns of family life, manifesting in new work patterns, chore allocations, and household tensions. Though men have taken an increased share of childcare and grocery shopping duties, reallocations are not nearly as stark as disruptions to work patterns might suggest, and families having to reallocate duties report greater tensions. Our results highlight tightened constraints budging up against stable and gendered patterns of intra-household cooperation norms. While the long-run consequences of the COVID-19 lockdown on family life cannot be assessed at this stage, we point toward the likely opportunities and challenges.

*‘‘Kitchen life is based on a musical rhythm, on a concatenation of movements, like dance steps, and when I speak of rapid gestures, it’s a female hand I think of, not my own clumsy sluggish movements, that’s for sure, always getting in the way of everybody else’s work. At least that’s what I’ve been told my life long by parents, friends -male and female- superiors, underlings and even my daughter these days. They’ve been conspiring together to demoralize me, I know; they think that if they go on telling me I’m hopeless they’ll convince me there’s an element of truth to the story. But I hang back on the sidelines, waiting for an opportunity to make myself useful, to redeem myself. Now the plates are all caged up in their little carriage, round faces astonished to find themselves standing upright, curved backs waiting for the storm about to break over them down there at the bottom of the tunnel where they will be sent off in exile until the cycle of cloudbursts, waterspouts and steam jet is over. This is the moment for me to go into action.’’ Italo Calvino, ‘‘La Poubelle Agree’’ in The Road to San Giovanni, pp. 58/59* ^1^

## Introduction

Frantically trying to limit the spread of the COVID-19 pandemic, governments worldwide imposed severe lockdown policies that suddenly changed the daily lives and routines of millions of people. This lockdown artificially created a fusion between the work and family life of men and women, who had to come to terms with their relative contribution to childcare and household chores. Such unexpected changes to the domestic division of labor fueled tensions and exacerbated pre-existing gender and socio-economic inequalities, and might lead to long-term changes in gender norms.

Through the lens of behavioral and gender economic models, augmented by language and discourse analysis, we view these lockdown policies as a requirement for citizens to cooperate with each other at multiple levels: on the one hand they need to cooperate with government in respecting lockdown measures themselves, and on the other they have to cooperate more within their households as the usual divisions between work, home, and school become blurred. It is important to understand how such cooperation has occurred as this has likely impacted households differently, depending on what happened to the livelihoods of household members and on the presence of children who need care and schoolwork help. For example, whilst the overwhelming evidence on the immediate *health* consequences of COVID-19 suggests that men have fared much worse than women, the emerging evidence on labor markets indicates that the impact has been stronger on sectors with high female employment shares and that women are more likely to be working in jobs that can be done from home and more likely to lose their jobs (Adams-Prassl et al., 2020 for the United Kingdom, the United States, and Germany; Alon et al., 2020 for the United States, Hupkau and Petrongolo, 2020 in the United Kingdom).

We study the personal and family consequences of this abrupt change in daily life via anonline survey in three of the most severely hit OECD countries – Italy, the United Kingdom, and the United States – during the height of the initial lockdowns. Looking at the reallocation of household chores following the lockdown, we find a dramatic increase in the proportion of shared childcare across all countries and increases in the sharing of most other household chores. The only exception is grocery shopping, which has instead become a more specialized task largely done by men. In all three countries we have surveyed, job loss or working from home when the partner is working outside are associated with a greater deviation from the *status quo* in terms of division of labor. These unexpected shifts in division of household tasks fueled an increase in tension within couples, suggesting that the disruption in who *did* what around the house often came into conflict with ideas about who *should* do various activities.

Documenting the extent to which family members have changed the work they do inside the household in response to lockdown is an important matter in both the short and long run, as this may dampen or amplify the effects of school closures on both children and their parents, women’s chances of returning to work, as well as mental health and family outcomes since domestic tensions can affect family stability (Ruppanner et al., 2018).

## Background Literature

Household bargaining models (Manser and Brown, 1980; McElroy and Horney, 1981; Lundberg and Pollak, 1993, 1994, 1996) predict that the division of tasks inside and outside of the household will be shaped by new labor market constraints, such as restrictions or expansions of working hours as well as the possibility of remote working and its relative flexibility. Updating this theoretical literature on household bargaining based on the expected results from the COVID-19 pandemic, Croda and Grossbard (2021) shows that the shifts taking place with the COVID-19 crisis suggested those with less bargaining power would acquire the majority of the additional domestic tasks. Bansak et al. (2021) indeed find that women living in United States states incentivizing stay-at-home parents (states with community property regimes or with homemaking provisions) were more likely to shift out of paid labor during school closures.

Besides changing external constraints, the COVID-19 crisis can be viewed as an information shock for both partners. This unanticipated shock may have revealed to partners their true (as opposed to expected) disutility from working from home, and the associated cost of sharing childcare and other household duties. Following the crisis, both partners might have updated their priors and re-bargained the division of household chores accordingly. As a result, we can expect: (1) an increase in household bargaining with its associated tension and stress; and (2) an increase in strategic behavior, with partners believing the situation to be temporary signaling a higher willingness to cooperate than would normally be the case but revealing their true colors by specializing at gendered tasks.

The burden of extra home production has fallen unequally on women with the potential for long-term negative impacts on their wages and job prospects, as well as potentially creating tensions within households. More positively, new ways of working – and the fact that many fathers are also now doing more – has been hailed as having the potential to help change gender norms and lead to a more equal allocation in some households in the longer term.

Although the expectation from the outset was that mothers would invest more of their own time and resources into home schooling, childcare, and domestic tasks than fathers (Sevilla and Smith, 2020) thus exacerbating existing inequalities (the parenting penalty literature has amply illustrated the impact of caring on women’s labor market outcomes Kleven et al., 2019), some hopeful voices were suggesting that fathers who were working from home or furloughed might actually change their preferences toward caring once they were exposed to large amounts of it (Alon et al., 2020).

## Materials and Methods

### Procedure, Participants, and Data Collection

In April 2020 we ran an online survey on a total of 3,157 adults (18--83 years old) and 235 children (4--18 years old). The survey was administered in three countries: the United States, the United Kingdom and Italy over the period 11--19 April, when our respondents had been in lockdown for between 5--6 weeks in Italy, 2--3 in the United Kingdom, and 1--4 in the United States depending on the respondent’s specific location. Rather than being a cross-cultural comparison study, these countries were chosen as they were among the worst affected OECD countries by COVID-19 (in its initial wave) in both reported COVID-19 deaths per capita,^2^ excess mortality during the pandemic^3^ and, according to OECD projections,^4^ in economic terms too.

The participants in the United States (949 adults and 42 children) and the United Kingdom (1,001 adults and 52 children) were recruited using an online survey collection tool^5^ which stratifies samples across age, sex, and ethnicity. The participants in Italy (1,207 adults and 141 children) were recruited primarily through social media and thus cannot be expected to constitute as representative a sample as those of the United States and United Kingdom.^6^ Of the 3,157 adult respondents, 2,526 indicated that they are cohabiting with either their partner or another adult during the quarantine period (1,034 in Italy, 800 in the United Kingdom, and 692 in the United States). This is the subset for which, when division of labor responses were provided, we measured and summarized the re-allocation of household tasks. Of these 2,526 cohabiting respondents, 893 indicated that they are also living with their children during the quarantine period (468 in Italy, 220 in the United Kingdom, and 205 in the United States).

### The Survey Instrument

All recruited participants were directed to a Google Forms survey which varied by country. The Italian participants completed a survey which was in Italian, and the United States and United Kingdom participants completed versions of the survey in English, with minor variations to account for language use and demographic questions which vary across the two countries. All versions of the full survey may be found at https://osf.io/upq5g/. Adults were asked 46 questions. For the purpose of this paper, we focus on the adults.

Our survey is a study of family life during the first lockdown, aimed at understanding how daily routine had been modified, how the division of labor within the household had changed, and how personal wellbeing, family tension, beliefs and aspirations, risk attitudes, and the willingness to cooperate within and outside of the household had been during this lockdown.

Questions asked about participants’ demographics, family status and living situation, as well as the ways in which the pandemic affected them and their households personally. This encompassed their health, wellbeing, employment situation, the allocation of labor within the household, tensions between household members, and anti-COVID prophylactic behaviors. Furthermore, to measure cooperation within couples, respondents took part in an incentivized Prisoners Dilemma game (Fehr and Gächter, 2002).

Most of our questions are adapted wholesale from two main validated sources: Understanding Society – and in particular the Understanding Society Coronavirus Study: March 2021 questionnaire – and the United Kingdom Labour Force Survey. Some questions particular to COVID-19 were not piloted, our intention was rather to get the surveys out as quickly as possible during the height of the first pandemic wave. The children’s survey was written following the model of the Youth Questionnaire by Understanding Society and is composed of 45 questions, among which we included one unincentivized risk elicitation question.

Many of the family dynamics we were interested in might have changed suddenly at the start of lockdown, so we asked subjects to describe their current as well as pre-pandemic work status, chore allocation, and levels of tension. This allowed us to implement a “pseudo-panel” design, in which we can investigate changes in the outcome for a participant, even though both are measured at the same moment in time.

## Results

### Summary Statistics

By April 2020, the impact of the virus was already sizable. We find that 17% of respondents in Italy, 11% of respondents in the United Kingdom, and 10% of respondents in the United States were directly affected by COVID-19 either because they were tested for it or knew someone who was infected. 15% of respondents in Italy, 20% of respondents in the United Kingdom, and 17% of respondents in the United States lost their job or were furloughed. On a psychological level, respondents showed high levels of anxiety^7^ (55% of respondents in Italy, 48% in the United Kingdom, and 43% in the United States reported to be anxious on the day prior to the survey), and low levels of happiness^8^ (13% of respondents in Italy and 24% in the United Kingdom and in the United States reported not being happy). Respondents clearly feel isolated, and most reported that one of the first things they would like to do once lockdown ends is to visit family and friends (78% of respondents in Italy, 77% of respondents in the United Kingdom, and 64% of respondents in the United States). 20% of respondents in Italy, 41% of respondents in the United Kingdom, and 47% of respondents in the United States reported that one of the first things they would like to do once lockdown ends is to go shopping.

Even while struggling with the personal and social toll imposed by the pandemic, individuals sustain high levels of cooperation. In terms of cooperation with lockdown measures, most people adopt the recommended protective measures such as washing hands (80% of respondents in Italy, 91% of respondents in the United Kingdom, and 90% of respondents in the United States), avoiding shaking hands (88% of respondents in Italy and 90% of respondents in the United Kingdom and the United States), keeping a safe distance from others (91% of respondents in Italy and 96% of respondents in the United Kingdom and the United States), and avoiding crowded places (83% of respondents in Italy, 92% of respondents in the United Kingdom, and 91% of respondents in the United States). Mask-wearing habits vary greatly by country, 84% of respondents in Italy, 13% of respondents in the United Kingdom, and 58% of respondents in the United States reporting that they wear a mask in public, reflecting the lack of a general consensus amongst governments and intergovernmental organizations on mask effectiveness at the time of the survey. A majority of respondents also follow more restrictive lockdown measures like limiting supermarket visits as much as possible (87% of respondents in Italy, 88% of respondents in the United Kingdom, and 89% of respondents in the United States), refraining from visiting friends (82% of respondents in Italy, 94% of respondents in the United Kingdom, and 82% of respondents in the United States), refraining from visiting relatives (82% of respondents in Italy, 92% of respondents in the United Kingdom, and 72% of respondents in the United States), and staying home except in case of emergency (78% of respondents in Italy, 47% of respondents in the United Kingdom, and 41% of respondents in the United States).

In terms of cooperation, 69% of respondents in Italy, 71% of respondents in the United Kingdom, and 75% of respondents in the United States are willing to cooperate with strangers who respect social distancing measures, whilst 21% of respondents in Italy, 14% of respondents in the United Kingdom, and 20% of respondents in the United States would cooperate also with strangers who do not respect measures. These results indicate a strong willingness to cooperate, but only with those who are deemed responsible and trustworthy. We furthermore tie households’ willingness to shift domestic labor allocations to cooperativeness. More cooperative households show propensity for a greater share of chores to be allocated toward the partner who experiences a relatively greater shift in time available to be spent at home – i.e., someone who has been furloughed when their partner has not. This indicates to us that the descriptive changes we see are not merely the utility-maximizing reallocations of a unitary household’s labor supply (Becker, 1965). Norms make behavioral patterns persistent (Young, 2015) but sometimes exogeneous shocks to behavior can cause long-term norm change (Bicchieri and Mercier, 2014). The COVID-19 pandemic has certainly constituted a great exogenous shock – we have yet to see which of its many disruptions persist.

### Allocation of Household Chores

In terms of household work, sharing of most duties increased during lockdown, but so did the burden on women. The proportion of *shared* childcare increased dramatically (17 percentage points in Italy, 8 percent in the United Kingdom and 11 percent in the United States), and for most other tasks (cleaning, cooking and gardening) sharing grew between 2 and 11 percentage points on average. The one exception is grocery shopping, which during lockdown became a more male-specialized task (sharing went down 16 percentage points in Italy, 12 percent in the United Kingdom and 9 percent in the United States). Overall, the burden of household chores on women increased, which is problematic as there are significant reductions in lifetime earnings associated with performing these activities (Folbre, 2018; Grossman, 2019; Chu et al., 2020).

When comparing reporting of household tasks, interesting gender discrepancies arise. There are gender differences in reported increases in both one’s own tasks (on average men report larger increases, driven by grocery shopping, childcare and cleaning), and in the partner’s tasks, with men both in the United Kingdom and the United States samples reporting they do more (although to a small extent) than what women say their partners do.

To understand the reallocation of tasks within the household, and the ensuing tension, it is important to first understand the *time constraints* faced by couples. Time constraints in our data are proxied by grouping individuals into three categories, according to their work status: working outside of the home (least time at home); working at home (moderate time at home); not working (most time available at home). Looking at the change in time constraints faced by respondents and their partners from before to during the lockdown, we can establish the potential for taking on more household work. We analyze the ‘‘shift in comparative advantage toward home production’’ by taking the difference between the respondents’ and their partners’ change in time constraints, in the spirit of a difference-in-differences^9^ approach (before vs. after the lockdown, self vs. partner). We focus on the perspective of individuals who saw an increase in time at home relative to their partners, for example people who started working from home during the lockdown while their partner kept on going to the office, or people who were laid off while the partner kept on working.^10^ As expected, those who lost their job report doing more now, while those who are still working report doing the same or less, especially in the case of women. The opposite is true for those whose partners lost their job, again especially for women. Similar results are found by Del Boca et al. (2020) who analyze the change in time use of a representative sample of 520 Italian women and find that the additional burden during lockdown has been greater on women than on men, regardless of the partner working arrangement, while men spend more time doing housework only when their partner continues to work outside of the household.

**FIGURE 1:**
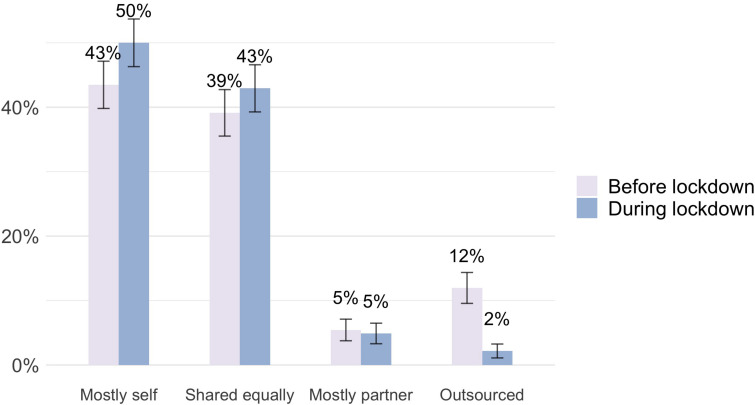
The division of childcare from before to during the lockdown, as reported by women who experienced a relative increase in time at home compared with their partner.

**FIGURE 2:**
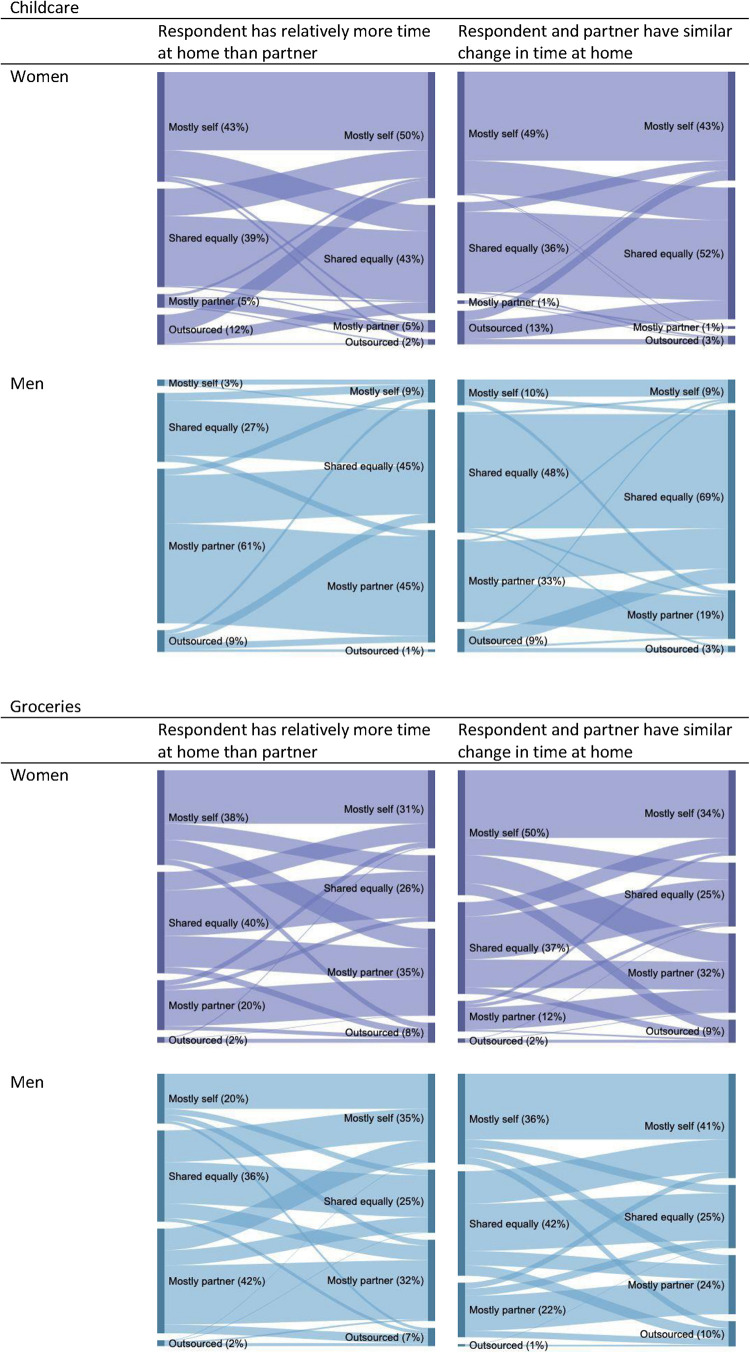
Changes in division of childcare and grocery shopping from before to during the lockdown, split by relative change in time at home. The above Sankey diagrams report changes in childcare and grocery shopping allocation from before the lockdown (left-hand side of each diagram) to during the lockdown (right-hand side of each diagram) for women and men respectively. The figures are split according to whether the respondent has relatively more time at home than their partner during the lockdown compared to before (left-hand side panel) or experienced a similar change in time at home as their partner following the lockdown (right-hand side panel). Source: online survey in Italy, United Kingdom, United States. For childcare, *N* = 476 (women) and 316 (men). For grocery shopping, *N* = 1,208 (women) and 873 (men).

Figures 1, 2 report changes in childcare and grocery shopping from before the lockdown to during the lockdown. The figures are split according to those who have more time at home during lockdown than before, relative to their partner, and those who do not. As an example, Figure 1 presents the division of childcare amongst women respondents who experienced a relative increase in time at home compared with their partner, that is women who spend more time at home during the lockdown than before, while their partner does not. The upper left panel of Figure 2 reports the same data as Figure 1 in Sankey diagram format. Sankey diagrams augment the before/after totals by showing the dynamics of the shifts in the division of labor. The width of the connecting segments in the Sankey diagram indicates the proportions of those who went from one category before to another after.

The left panel of Figure 2 includes those who have more time at home during lockdown than before relative to their partner (for example because they started working from home while their partner still works from the office) while the right panel includes those whose time constraints relative to their partner remain unchanged (for example because both used to work outside and both started working from home during lockdown).

For childcare, both men and women who saw a shift in comparative advantage toward home production take on more of this responsibility themselves compared to before. This same pattern, though slightly less pronounced, holds true across most other household work (see Supplementary Figure 1). However, when we look at grocery shopping, men are taking on more of it, while women less, regardless of their relative job status. This shift to men doing the shopping occurs across all households, including the ones where we would predict otherwise based on available time at home. The fact that relative time constraints are not predictive of who is doing grocery shopping suggests that the importance of time availability is outweighed by other factors such as risk perceptions, the unskilled nature of the task, and gender norms. For example, a possible interpretation of this finding is that men are more willing to take the risk (and possibly the pleasure) of going out of the house to buy food, or conform to the gender norms pertaining to the role of men as hunters or connectors between the domestic and public sphere. Gender norms are known to be related to a range of family, economic, and educational outcomes (Inglehart and Norris, 2003; Seguino, 2007; Guiso et al., 2008), and are quite different across the three countries that we surveyed.^11^

Additional evidence supporting the notion that shifting time availability is predictive of some – but not all – variation in household task reallocation is shown in Table 1. Here we report the marginal coefficients from ordered probit regressions using time constraints and cooperation with the partner to predict the change in household tasks following the lockdown. The outcome variable is coded such that a higher number is indicative of less involvement.^12^ We see that having relatively more time at home is always related to greater involvement in household chores (a negative coefficient), slightly more for men than women, although often the relationship is small. Specifically, men who experience relatively more time at home compared to their partners take on a greater share of childcare, as well as a greater share of grocery shopping, though this latter effect is smaller as we observe men taking on more grocery shopping duties regardless of their change in relative time at home. Only a few women are seen to take on a greater share of grocery shopping when they experience an increase in available time at home relative to their partners. Women also take on more cleaning duties when they experience an increase in relative time at home. Interestingly, whether respondents would be willing to cooperate with their partners in the Prisoner’s Dilemma game is also predictive of taking on more household responsibilities during lockdown, particularly men taking on more childcare and women doing more cleaning. Controlling for propensity to cooperate with one’s partner does not substantially change the estimated predictive power of experiencing a relative shift in time at home, suggesting independent contributions to the respondents’ willingness to reallocate household chores. Few movements in the allocation of cooking, laundry, or gardening duties are predicted. Supplementary Figure 2 plots the coefficients from the ordered probit regressions in Table 1, as well as estimates of these coefficients in which the Italian respondents are weighted by similarity to the representative United States and United Kingdom samples.^13^ Estimates are broadly similar whether or not we account for selection in the Italian sample.

**TABLE 1:** Ordered probit regressions predicting changes in family chore allocations.

*Men*												

	(1)	(2)	(3)	(4)	(5)	(6)	(7)	(8)	(9)	(10)	(11)	(12)

	Childcare		Groceries		Cooking		Cleaning		Laundry		Gardening	
Relatively	−0.401**	−0.393**	−0.240***	−0.240***	–0.060	–0.060	–0.066	–0.062	–0.072	–0.072	–0.076	–0.076
More time	(0.177)	(0.178)	(0.091)	(0.177)	(0.105)	(0.105)	(0.112)	(0.112)	(0.121)	(0.121)	(0.122)	(0.122)
Cooperate		−0.309*		0.017		–0.023		–0.158		–0.012		–0.005
w/partner		(0.184)		(0.099)		(0.110)		(0.116)		(0.127)		(0.125)

Survey FE	Yes	Yes	Yes	Yes	Yes	Yes	Yes	Yes	Yes	Yes	Yes	Yes
N	214	214	646	646	646	646	646	646	646	646	646	646

***Women***												

	**(1)**	**(2)**	**(3)**	**(4)**	**(5)**	**(6)**	**(7)**	**(8)**	**(9)**	**(10)**	**(11)**	**(12)**

	**Childcare**		**Groceries**		**Cooking**		**Cleaning**		**Laundry**		**Gardening**	

Relatively	–0.198	–0.197	−0.168**	−0.169**	–0.006	–0.004	−0.169*	–0.163	–0.169	–0.171	–0.017	–0.022
More time	(0.153)	(0.153)	(0.079)	(0.079)	(0.098)	(0.098)	(0.105)	(0.105)	(0.109)	(0.186)	(0.010)	(0.100)
Cooperate		–0.207		–0.017		0.055		0.174*		–0.071		–0.157
w/partner		(0.151)		(0.080)		(0.105)		(0.108)		(0.113)		(0.109)

Survey FE	Yes	Yes	Yes	Yes	Yes	Yes	Yes	Yes	Yes	Yes	Yes	Yes
N	305	305	849	849	849	849	849	849	849	849	849	849

The coefficients are marginal effects from an ordered probit regression. Standard errors in parenthesis. *Indicates *p*-value < 0.10; **p-value < 0.05; ***p-value < 0.01. *Outcome* variable is the first-difference (during the lockdown minus before) in self-reported allocation of several household tasks, with 2 corresponding to “Mostly partner,” 1 to “Shared equally,” and 0 to “Mostly self.” All other answers (“paid help/deliveries” or “prefer not to say”) are coded as missing. *Relatively more time* is an indicator variable for having relatively more time at home than the partner during the lockdown compared to before [constructed as a difference-in-differences between the time available at home because of job status during the pandemic vs. before (first diff.) and of the respondent vs. the partner (second difference)]. *Cooperate with partner* is an indicator variable for willingness to cooperate with the partner in a Prisoner’s Dilemma game. *Controls* include country fixed effects and polynomial in age. Source: online survey in Italy, United Kingdom, United States.

### Family Cooperation and Tensions

So far, we have shown that the lockdown led to substantial reallocation of household chores, following not only changes in time constraints, but also individual propensity to cooperate with the partner and task-specific gender norms. Next we ask: is this reallocation of tasks conducive to more or less harmony within the couple? To investigate the potential consequences of an uneven reallocation of chores, we examine the respondent’s report on tensions about the division of household labor, quarrels before and during the lockdown, and the language used to discuss these issues.

Marked gender differences are present when looking at tension over the division of household tasks and general wellbeing. Tensions in the household are reported in all countries, with women generally reporting higher household tensions than men. Some household tension^14^ is reported by 28% of men and 43% of women amongst respondents in Italy, 28% of men and 37% of women amongst respondents in the United Kingdom, and 32% of both men and women amongst respondents in the United States. Child respondents report household tensions more frequently than adults, with 67% of children from the Italy sample and 64% of children from the United Kingdom and United States samples reporting significant household tension. In line with national surveys of wellbeing over the same period, most respondents report higher anxiety and lower instantaneous wellbeing relative to overall life satisfaction and sense of leading a worthwhile life, with women reporting consistently higher anxiety and lower wellbeing than men in both Italy and the United Kingdom, while the averages are closer for women and men in the United States sample. Average life satisfaction is 5% lower amongst women than men in the Italy sample, 1% lower in the United Kingdom sample, and less than 1% lower in the United States sample. Instantaneous anxiety, on average, is 19% higher among women than men in the Italy sample, and 12% higher among women relative to men in the United Kingdom and United States sample.^15^ These findings align with those in the United Kingdom and United States indicating that women, and mothers in particular, experienced a markedly larger decline in wellbeing than men during the pandemic (Zhou et al., 2020; Prados and Zamarro, 2021). When asking questions directly to children, we find that those with above-average assessments of their school, their teachers, how hard they work, and how well they perform consistently report higher wellbeing and instantaneous wellbeing than children with below-average assessments, as do those who report using social media less than an hour both during quarantine and before.

**FIGURE 3:**
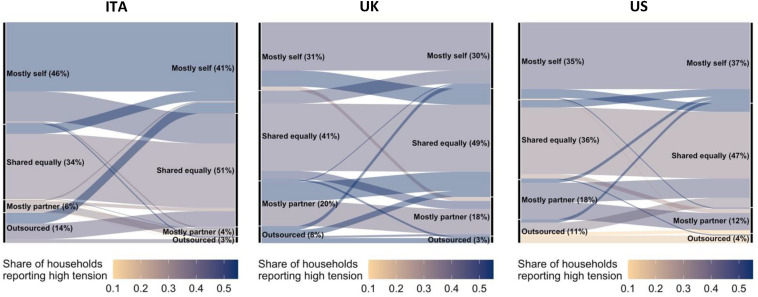
Changes in division of childcare from before to during the lockdown, colored by share of households reporting high tension. The above Sankey diagrams report changes in childcare allocation from before the lockdown (left-hand side of each diagram) to during the lockdown (right-hand side of each diagram) for each of the countries surveyed. Diagram flows are color-coded by the share of respondents reporting high household tensions specifically related to the allocations of household tasks. Darker lines correspond to subsets with higher reported household tensions, and are useful in capturing the effect of task reallocation in lockdown. Source: online survey in Italy, United Kingdom, United States; *N* = 893.

**FIGURE 4:**
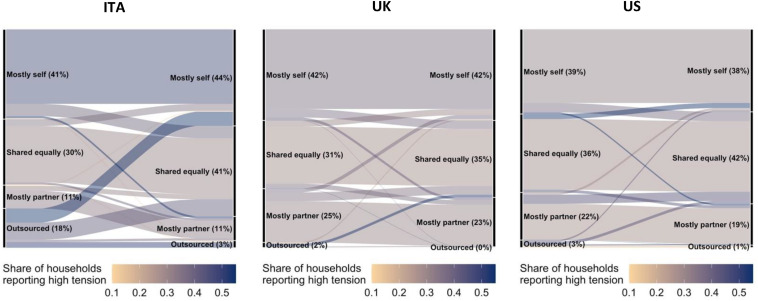
Changes in division of cleaning from before to during the lockdown, colored by share of household reporting high tension. The above Sankey diagrams report changes in cleaning allocation from before the lockdown (left-hand side of each diagram) to during the lockdown (right-hand side of each diagram) for each of the countries surveyed. Diagram flows are color-coded by the share of respondents reporting high household tensions specifically related to the allocations of household tasks. Darker lines correspond to subsets with higher reported household tensions, and are useful in capturing the effect of task reallocation in lockdown. Source: online survey in Italy, United Kingdom, United States; *N* = 2,527.

To understand how these changes in wellbeing are related to reallocation of household tasks, the Sankey diagrams in Figures 3, 4 represent how the allocation of childcare and cleaning changed from before to during the lockdown, for Italy, the United Kingdom, and the United States respectively, with flows color-coded based on the level of household tension reported by respondents specifically related to the allocations of household tasks.^16^ Darker lines indicate higher levels of reported tension. Considering for example childcare, across all samples, the respondents more likely to report the lowest level of tension in the household are those who share childcare, alongside those who report that their partner is mostly doing it and, only in the United States sample, those who outsource it. This observation aligns with the finding from a study in the United States that insufficient support with childcare has been a key driver of conflict amongst couples with young children during the lockdown (Calarco et al., 2020). The respondents who report high levels of tensions vary by country. Respondents in Italy who report the highest tension are those who either continue to be solely responsible for childcare or saw a reallocation of childcare to themselves, compared to a previous shared or outsourced provision. This is different from the United Kingdom case, where the highest tensions are reported by respondents who are now sharing more of the childcare than before the lockdown, regardless of whether they were previously solely responsible or their partner was. The United States sample is somewhat in between, with highest tensions reported by both those who saw an increase in their own load and those who were previously solely responsible and started sharing during the lockdown.

When considering other household activities, we again find that respondents reporting the lowest levels of tension are those who report sharing tasks. High levels of tension are related to deviations from the *status quo*, and not just changes that increase one’s own load, but also those that shift tasks away from oneself and to the partner. These patterns of low tension when sharing and high tension when changing allocations are clearly illustrated by the diagrams displaying changes in the allocation of cleaning in Figure 4 (see Supplementary Figures 3–6 for the other tasks).

Additional evidence supporting the notion that changes in allocation of household tasks is predictive of higher tension is shown in Table 2. Via an OLS regression, we find that changing the usual allocation of any household task during the lockdown is related to higher levels of tension. Higher tension is particularly predicted by changes in grocery shopping, cleaning, and childcare duties (see Supplementary Tables 2, 4), while the association with changes in cooking and gardening chores is smaller and less precisely estimated. To give an idea of the magnitude, the association between tension and changing who is in charge of groceries or cleaning because of the lockdown is between one third and one half of the association between tensions during the lockdown and having a child present in the household (see Supplementary Table 1a).^17^ Except for cooking, the strong association between changing tasks and tension is robust to the inclusion of detailed controls for the respondent’s and their partner’s job status, as well as personal characteristics such as cooperation, risk seeking, mental health and wellbeing (see columns 2 and 3 of Table 2 and Supplementary Tables 1–4). Furthermore, similar patterns can be found by using an indicator of higher levels of quarreling during the lockdown as outcome variable (see Supplementary Tables 3, 4).^18^

**TABLE 2:** OLS regression predicting tension due to change in allocation of household tasks.

	(1)	(2)	(3)	(4)

	Tension over the division of household tasks			
Changed division: grocery	0.284**	0.264**	0.246**	0.324
	(0.114)	(0.119)	(0.115)	(0.210)
Ch. Grocery × fem				–0.119
				(0.251)
Changed division: clean	0.472***	0.440***	0.390**	0.491*
	(0.161)	(0.163)	(0.155)	(0.259)
Ch. clean × fem				–0.157
				(0.321)
Changed division: cook	0.117	–0.002	0.045	0.078
	(0.183)	(0.183)	(0.178)	(0.279)
Ch. cook × fem				–0.070
				(0.363)
Changed division: gardening	0.113	0.200	0.164	0.337
	(0.175)	(0.189)	(0.177)	(0.364)
Ch. gardening × fem				–0.241
				(0.416)
Job status	No	Yes	Yes	Yes
Personal characteristics	No	No	Yes	Yes
N	2348	2121	2120	2111

Coefficients from an OLS regression. Robust standard errors in parenthesis. *Indicates *p*-value < 0.10; **p-value < 0.05; ***p-value < 0.01. *Outcome* variable is self-reported answer to the question ‘Are you experiencing tensions over the division of work to do in the household at the moment?’ on a scale from 0 (no tension at all) to 10 (a lot of tension). *Changed division*: indicator equal to one if the division of the household task is different during the lockdown than before, and zero otherwise (i.e., indicator for the diagonal flows in the Sankey diagrams). *Ch. × fem*: interaction between the indicator for changed division of household labor and female respondent. *Demographic controls*: cubic polynomial in age and indicator for presence of children in the household. *Job status*: controls for respondent and partner’s job status, including indicators for working remotely (omitted category); working outside of home (both as essential workers and non-essential workers); work for a family business; government-sponsored training scheme; apprenticeship; employed with other paid work; self-employed; furlough; temporary leave (e.g., maternity leave or ill); student; homemakers; retired. *Personal characteristics*: controls for cooperating with the partner in a Prisoner’s Dilemma game; indicator for risk-seeking behaviors reported in reasons to leave home (see friends, tired of being in the home, getting bored, getting some adrenaline, exercising free will); self-reported life satisfaction; living a worthwhile life; happiness; anxiety; frequency talking with family or friends; indicator for wanting to buy a gift to the partner when lockdown ends. Source: online survey in Italy, United Kingdom, United States.

Gender differences in the relationship between tensions and changes in allocation of household tasks are not pronounced. As shown in column 4 of Table 2 (and Supplementary Tables 1–4), gender differences in this association are usually small, and often noisily estimated. Exceptions are changes in who is responsible for gardening, which is twice as strongly associated with tension when the respondent is male (0.337 for males, 0.337–0.241 = 0.096 for females, but the difference is still not statistically significant), and changes in childcare (which is strongly associated with tension when the respondent is male, almost uncorrelated if female, see Supplementary Table 2, column 4).

These results are important as tensions can impact family stability: divorce filings were reported to be on the rise in Wuhan^19^ and family dynamics can be altered by calamities and natural disasters: divorces increased in New York after 9/11 and marriage, birth, and divorce rates increased in the year following Hurricane Hugo in 1989 in the 24 counties of South Carolina that were declared disaster areas compared with the 22 other counties in the state (Cohan and Cole, 2002; Cohan et al., 2009). In our sample, 21 of 2,607 respondents with partners declare they want a divorce when quarantine ends. Our survey instrument was not designed to investigate domestic violence and the nature of our sample and its collection mode would probably have excluded vulnerable families where this issue would be more prevalent, but it is important to note that lockdown has been linked to domestic violence (Peterman et al., 2020), and the inability to meet financial obligations and maintaining social ties is likely to increase family stress and domestic violence (although Beland et al., 2020, do not find strong evidence in this regard).

### Talking Through It

Communication difficulties play a vital role in marriage unhappiness and communications-related issues are cited much more often as causes for divorce than external issues, including economic ones (Thompson, 2008). To better understand potential issues with communication, we analyzed the language that respondents used to answer open ended questions to our survey. When it comes to the language used to address tensions arising from the establishment of a new routine and allocation of household tasks during the lockdown, we find markedly different styles by gender and, to a lesser extent, by country. In all three countries, women are more likely than men to voice their concerns in our survey. When addressing the disagreement (about half the women in our sample prefer to say nothing) women talk about their expectations, dissatisfaction, and anger. Men’s preferred strategy is to say nothing, and when they do, they do so to signal there is not a big problem and no routine has been established, often because it does not seem to be needed.

**FIGURE 5:**
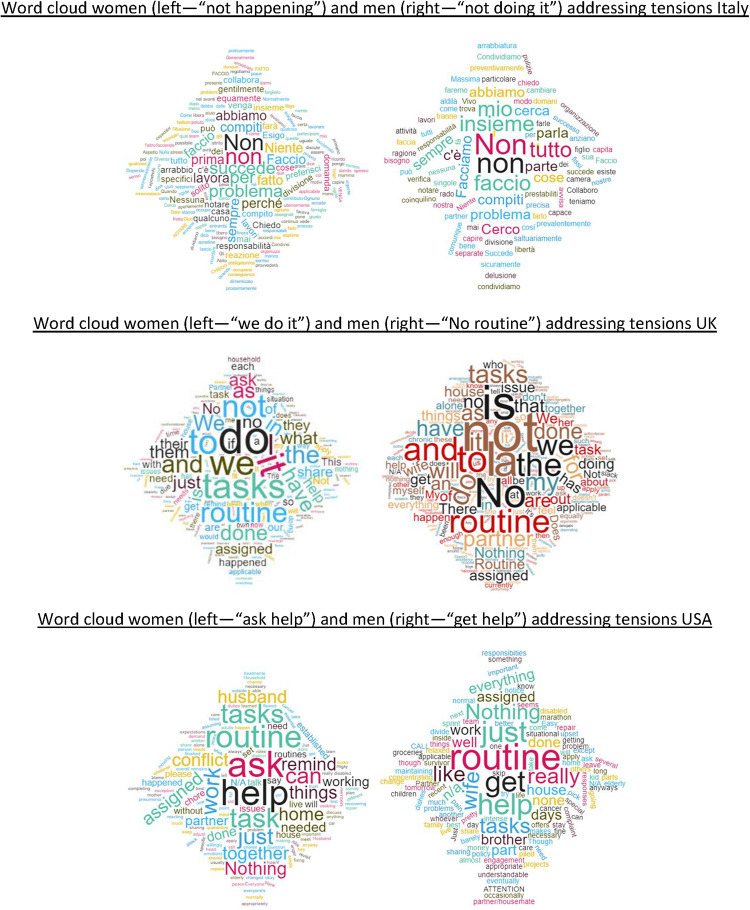
Word clouds from the open answers to the question regarding tension on the division of assigned household tasks.

The word clouds in Figure 5 show the language used by female and male respondents in each country.

This gender difference in the use of language to talk about tensions can be interpreted as a reflection of the gendered expectations in terms of role divisions, and might further reinforce such roles. Household work and the related communications are seen as a female domain and not a space for men to engage in conversations. The ‘proper’ workplace, and not the household, is the place for men to communicate. Also, women are socially expected to express emotions and hence are possibly more likely to open up about their frustrations as opposed to men who are expected to be more restrained (Lakoff, 1975; Tannen, 1990; Sunderland, 2004; Jaworska and Ryan, 2018).

## Conclusion

Our study finds a dramatic increase across Italy, the United Kingdom, and the United States in the proportion of shared childcare, and increases in the sharing of most other tasks, with the exception of grocery shopping which instead became a more specialized task done largely by men during the lockdown. In all three countries we surveyed, the reallocation of household tasks mirrors the relative changes of job status within the couple: respondents who lost their job (while their partners did not) or who are working from home (while their partners kept on working outside of the house) are shouldering a greater share of household chores. The opposite is true for those whose partners lost jobs (but not them). Thus, asymmetric changes in job situations are strongly associated with a deviation from the *status quo* in terms of division of labor.

The specialization pattern we find, with women doing more of everything and men doing more shopping, is corroborated by a range of studies carried out during the crisis. In the United States, Carlson et al. (2020) find that both parents report devoting more time to housework, with substantial increases in the sharing of both childcare (from 50 to 60%) and household tasks (from 38 to 53%). Such increases in sharing, however, are slightly disproportionate: in childcare, mothers do more of the homework supervision and fathers more of the playtime; in household tasks, fathers especially increased time devoted to grocery shopping. Parents also disagree on how much fathers actually do: 42% of fathers report an increase in housework time, 45% report more time in the care of young children overall, and 43% report more total care of older children, while only 25, 34, and 20% of mothers respectively say their partners did so. Sevilla and Smith (2020) show that United Kingdom families with young children have been doing the equivalent of a working week in childcare, with women doing the greater share and a reduction in the gender childcare gap, with men’s increases very sensitive on their employment status (whether they work from home or have been furloughed or lost their job). In Spain, Farré et al. (2020) show increases in women’s loads and a similar pattern of men specializing at grocery shopping, possibly, they argue, because it is a relatively easy, out-of-household task and perceived as carrying more risk.

We must also caution that while our United Kingdom and United States samples are representative on a few sociodemographic variables (age, ethnicity, gender), we have obviously surveyed a segment of the population with stable access to the internet, as well as time availability to complete the survey. We are therefore unlikely to have sampled those families with the greatest tensions or sharpest time constraints. More work must be done to assess the needs of the most vulnerable families, especially since their wellbeing and health are most at risk from the COVID-19 crisis.

As with much of the COVID-19 crisis, it is early days to speculate on the durability of these changes. However, there is some hope that more sharing of childcare and household work might be the silver lining on the cloud of adverse occupational effects that women are set to face: Alon et al. (2020) and Hupkau and Petrongolo (2020) speculate that this pandemic and the consequent reallocation of household chores may lead to a change of work and gender norms similar to that experienced with paternity leave introductions. However, these increases in sharing are not documented across all households, but rather among respondents who also report low tensions, and we might therefore be seeing a very partial silver lining, with women in some households experiencing multiple in- and out-of-household shocks.

There is, as yet, no direct evidence on the impact of COVID-19 on gender norms. In many households, women are doing more childcare, and pre-existing norms may become entrenched. But some households, particularly those where men are not working, are now experiencing a more equal gender division and this may lead to longer-term positive changes, particularly if they are combined with new ways of working (more working from home). Sevilla and Smith (2020) report that 28% of those who are currently working from home did not previously do so.

Even although the pandemic is forcing men to participate more in house work, many still do so by exercising their freedom to choose the more pleasant tasks, deciding how to contribute through gender-tinted lenses. The disaster literature suggests alternative scenarios for the short and the long run in terms of changes in the division of labor: Peek and Fothergill (2008) relay how the gendered division of labor may be even more pronounced in disasters, with women cast as nurturers and men as protectors, but also cite studies conducted on hurricane Andrew in the 1990s that found that, while gender roles were suspended and readapted during the crisis, they then reverted to previous arrangements (Alway et al., 1998) largely due to external constraints related to labor market forces and availability of childcare. Some evidence from paternity leave policies suggests that temporary changes can have longer-term effects on social norms, shown by increases in the time that fathers spend in household activities, including childcare (Farré and González, 2019; Patnaik, 2019). In the United Kingdom, data from Understanding Society show however that with the easing of restrictions toward the end of 2020, the share of fathers working positive hours had recovered close to their pre-pandemic rates, but for mothers, particularly single mothers, they continued to lag (Harkness, 2021).

A feature of the COVID-19 lockdown is that most of the work that was still happening, and all of the childcare, have moved into homes. This forced fusion of work and family life means that men at the very least witnessed, if not shared, the demand to be available for both work and family, typically experienced more acutely by working mothers. We do not yet know whether this will be sufficient to generate the changes in workplace and household culture necessary to create more balanced allocations of both paid and unpaid work (Grossbard-Schectman, 1993; Goldin, 2014; Folbre, 2018), but the differences we find in levels of tension across households suggest this will not be a smooth or an evenly distributed transition.

Two things are distinctive about COVID-19 lockdowns. The first is the scale of the demand-side shock. The changes have been profound. The total amount of childcare being done at home is of a completely different order of magnitude higher than usual because of the closure of almost all formal childcare. The impact has been across the board, affecting all families, meaning that almost all men have increased the quantity of childcare they do.

The second difference is that this is not a deliberate policy to promote a more equal distribution of childcare: changes in the division of labor are unintended consequences of measures to stop the virus from spreading. The changes in the division of household tasks that have been brought about may need to be recognized and reinforced to have longer-term effects.

## Data Availability Statement

The datasets presented in this study can be found in online repositories. The names of the repository/repositories and accession number(s) can be found below: https://osf.io/upq5g/.

## Ethics Statement

The studies involving human participants were reviewed and approved by University of Reading, School of Politics, Economics and International Relations Ethics Committee. Written informed consent to participate in this study was provided by the participants’ or their legal guardian/next of kin.

## Author Contributions

SB, PB, and JV generated the Sankey diagrams of household task allocations. PB, JV, and MDG generated the household tension diagrams. SJ generated the word cluster diagrams. All authors contributed to the design of the study and to the writing of the manuscript.

## Conflict of Interest

The authors declare that the research was conducted in the absence of any commercial or financial relationships that could be construed as a potential conflict of interest.

## Publisher’s Note

All claims expressed in this article are solely those of the authors and do not necessarily represent those of their affiliated organizations, or those of the publisher, the editors and the reviewers. Any product that may be evaluated in this article, or claim that may be made by its manufacturer, is not guaranteed or endorsed by the publisher.

## Supplementary Material

The Supplementary Material for this article can be found online at: https://www.frontiersin.org/articles/10.3389/fpsyg.2021.687570/full#supplementary-material

## Footnotes

1 All of the code and the (de-identified) data to reproduce the results presented in this paper can be found here https://osf.io/upq5g/

2
https://coronavirus.jhu.edu/map.html

3
https://www.economist.com/graphic-detail/2020/04/16/tracking-covid-19-excess-deaths-across-countries

4
http://www.oecd.org/economic-outlook/june-2020/

5
https://www.prolific.co/

6 We use Inverse Probability Weighting (IPW) to reproduce our regression analyses while controlling for selection bias in the Italian sample. See the Supplementary Materials. Results are broadly consistent with unweighted estimates. For ease of interpretation we focus on our sample as it is, without weights.

7 Reporting 5 or more on a scale from 0 (“not at all”) to 10 (“completely”) to the question “How anxious did you feel yesterday?”

8 Reporting less than 5 on a scale from 0 (“not at all”) to 10 (“completely”) to the question “How happy did you feel yesterday?”

9 Ours is an event study. We emphasize that while the COVID-19 pandemic was an exogenous shock, families’ labor-force responses to it are endogenous. I.e., an essential worker may nevertheless have chosen to leave their job in order to look after family. We cannot control for the unobserved reasons why people changed their labor market status, and do not make causal claims about how work arrangements affect the division of household labor. Our purpose is rather to document how these changed due to the COVID-19 pandemic.

10 Only one member of the couple responds to the survey, and reports both their own and their partner’s job status before and during the lockdown. To keep the perspective of the partner with more time available at home, sometimes the answers are swapped. That is, if the respondent has relatively more time at home during the lockdown than their partner, we keep the answers related to the respondent; if the opposite occurs, we look at the answers related to the respondent’s partner.

11 The United States and United Kingdom are both ranked 15th in the Gender Development Index of the United Nations Development Programme (http://hdr.undp.org/en/content/gender-development-index-gdi), and the labor force gender participation gap is smallest in the United Kingdom (10.6 percentage points in 2018 according to the OECD (https://stats.oecd.org/index.aspx?queryid=54751), followed by the United States with 12% and Italy with 18.3%). However, the last WEF report on gender equality (http://www3.weforum.org/docs/WEF_GGGR_2020.pdf) indicates that the representation of women on company boards is highest in Italy at 34% (this was mandatory for listed companies since 2012), followed by the United Kingdom at 27.2%, and the United States at 21.7%; politics is also somewhat different with 30% of women MPs the United Kingdom, 19.1% in the United States, and 31% in Italy.

12 Specifically, the outcome variable is the first difference (during vs. before the lockdown) of self-reported allocation of several household tasks, coded such that 2 corresponds to “Mostly partner,” 1 to “Shared equally,” and 0 to “Mostly self.”

13 Observations are weighted using an Inverse Probability Weighting (IPW) method as suggested by, e.g., Hernán et al. (2004); Howe et al. (2016). We run a logit regression where the outcome variable is an indicator for being part of the Italian sample and the explanatory variables are gender, age, age squared, age cube, presence of children, and several indicator variables for the job status (essential job, furloughed, freelance, family job, on leave, homemaker, in education, retired). The predicted probability $\hat{p}$ from this logit regression is used to construct IPW weights: $\left(1-\hat{p}\right)\,/\,\hat{p}$ for the Italian sample and 1 for the rest.

14 Reporting 3 or more on a scale from 0 (“no tension at all”) to 10 (“a lot of tension”) to the question “Are you experiencing tensions over the division of work to do in the household at the moment?”

15 This is calculated by computing simple averages of the 1–10 scale responses for the wellbeing variables from each group and then the percent increase/decrease in this average going from the male group to the female group in each country.

16 After the questions about the division of tasks, we asked “Are you experiencing tensions over the division of work to do in the household at the moment?”, with possible answers from 0 (“no tension at all”) to 10 (“a lot of tension”). Similar results can be found by color-coding the flows by answers to the question “How often do you and your partner/flatmate quarrel?”

17 Inverse Probability Weighted (IPW) regressions which attempt to control for selection in the Italian sample may be found in Supplementary Table 1b. The weights are calculated as above.

18 Since both the outcome variable (change in frequency of quarreling) and the main regressors (change in allocation of household tasks) are first-differences from during to before the lockdown, this analysis is similar to fixed-effect regression holding fixed time-invariant individual unobservables.

19
https://www.globaltimes.cn/content/1181829.shtml
