# Supplementary material for: Family Life in Lockdown
Source: Front Psychol. 2021 Aug 4;12:687570. doi: 10.3389/fpsyg.2021.687570 (PMC8371690; doi:10.3389/fpsyg.2021.687570)
Supplement: Supplementary file 1 [file Data_Sheet_1.docx]

Family Life in Lockdown

30 June 2021

*Pietro Biroli^[[1]](#footnote-1)^, Steven Bosworth^[[2]](#footnote-2)^, Marina Della Giusta^2^,*

*Amalia Di Girolamo^[[3]](#footnote-3)^, Sylvia Jaworska^2^, Jeremy Vollen^1^*

# Appendix

## Supplementary Tables

**Supplementary Table 1a: OLS regression predicting tension due to change in allocation of household tasks (all coefficients)**

|  | (1) | (2) | (3) | (4) |
| --- | --- | --- | --- | --- |
|  | Tension over the division of household tasks | | | |
| Changed division: grocery | 0.284** | 0.264** | 0.246** | 0.324 |
|  | (0.114) | (0.119) | (0.115) | (0.210) |
| Ch. grocery x fem |  |  |  | -0.119 |
|  |  |  |  | (0.251) |
| Changed division: clean | 0.472*** | 0.440*** | 0.390** | 0.491* |
|  | (0.161) | (0.163) | (0.155) | (0.259) |
| Ch. clean x fem |  |  |  | -0.157 |
|  |  |  |  | (0.321) |
| Changed division: cook | 0.117 | -0.002 | 0.045 | 0.078 |
|  | (0.183) | (0.183) | (0.178) | (0.279) |
| Ch. cook x fem |  |  |  | -0.070 |
|  |  |  |  | (0.363) |
| Changed division: gardening | 0.113 | 0.200 | 0.164 | 0.337 |
|  | (0.175) | (0.189) | (0.177) | (0.364) |
| Ch. gardening x fem |  |  |  | -0.241 |
|  |  |  |  | (0.416) |
| Age | -0.013* | -0.011 | -0.006 | -0.006 |
|  | (0.007) | (0.007) | (0.007) | (0.007) |
| Age squared | 0.000 | 0.000 | 0.000 | 0.000 |
|  | (0.000) | (0.000) | (0.000) | (0.000) |
| Age cubed | -0.000* | -0.000 | -0.000 | -0.000 |
|  | (0.000) | (0.000) | (0.000) | (0.000) |
| Children present | 1.022*** | 0.997*** | 1.094*** | 1.095*** |
|  | (0.122) | (0.128) | (0.122) | (0.123) |
| Female | 0.176* | 0.249** | 0.142 | 0.221* |
|  | (0.106) | (0.116) | (0.113) | (0.132) |
| UK survey | 0.039 | -0.019 | -0.362** | -0.368** |
|  | (0.128) | (0.141) | (0.147) | (0.146) |
| US survey | -0.016 | -0.017 | -0.353** | -0.354** |
|  | (0.130) | (0.140) | (0.144) | (0.145) |
| Work outside home as essential worker |  | 0.082 | 0.079 | 0.079 |
|  |  | (0.188) | (0.179) | (0.179) |
| In furlough |  | 0.176 | 0.145 | 0.139 |
|  |  | (0.257) | (0.235) | (0.235) |
| Self-employed or freelance |  | -0.318** | -0.336** | -0.341** |
|  |  | (0.142) | (0.132) | (0.132) |
| Working for your own or family business |  | -0.086 | -0.041 | -0.061 |
|  |  | (0.373) | (0.339) | (0.340) |
| Ill, maternity leave, on holiday, or temp leave |  | 0.174 | 0.137 | 0.143 |
|  |  | (0.360) | (0.352) | (0.354) |
| Full time responsibility for family and home |  | -0.263 | -0.248 | -0.256 |
|  |  | (0.202) | (0.191) | (0.191) |
| In education |  | 0.092 | -0.007 | -0.014 |
|  |  | (0.302) | (0.301) | (0.301) |
| Retired |  | -0.420** | -0.393** | -0.393** |
|  |  | (0.191) | (0.181) | (0.180) |
| Partner works outside home as essential worker |  | 0.075 | 0.079 | 0.073 |
|  |  | (0.155) | (0.147) | (0.147) |
| Partner in furlough |  | 0.308 | 0.248 | 0.242 |
|  |  | (0.228) | (0.215) | (0.215) |
| Partner self-employed or freelance |  | 0.242 | 0.245* | 0.248* |
|  |  | (0.153) | (0.145) | (0.146) |
| Partner working for your own or family business |  | 0.467 | 0.437 | 0.444 |
|  |  | (0.518) | (0.483) | (0.483) |
| Partner ill, maternity leave, on holiday, or temp leave |  | 0.565 | 0.554 | 0.560 |
|  |  | (0.477) | (0.471) | (0.471) |
| Partner full time responsible for family and home |  | 0.119 | 0.100 | 0.103 |
|  |  | (0.257) | (0.242) | (0.242) |
| Partner in education |  | 0.589* | 0.736** | 0.751** |
|  |  | (0.356) | (0.352) | (0.355) |
| Partner retired |  | 0.268 | 0.292* | 0.292* |
|  |  | (0.187) | (0.177) | (0.177) |
| Cooperate with Partner in the dilemma game |  |  | -0.034 | -0.031 |
|  |  |  | (0.106) | (0.106) |
| Risk-seeking |  |  | 0.570*** | 0.570*** |
|  |  |  | (0.176) | (0.176) |
| Life satisfaction |  |  | -0.080** | -0.080** |
|  |  |  | (0.036) | (0.036) |
| Life worthwhile |  |  | -0.066* | -0.067* |
|  |  |  | (0.035) | (0.035) |
| Happy |  |  | -0.105*** | -0.103*** |
|  |  |  | (0.037) | (0.037) |
| Anxious |  |  | 0.135*** | 0.136*** |
|  |  |  | (0.021) | (0.021) |
| Frequency talking with friends/family in lockdown |  |  | -0.039 | -0.039 |
|  |  |  | (0.043) | (0.043) |
| Wants to gift partner |  |  | -0.452*** | -0.451*** |
|  |  |  | (0.172) | (0.172) |
| Constant | 2.182*** | 2.020*** | 3.295*** | 3.242*** |
| Demographic controls | Yes | Yes | Yes | Yes |
| Job status | No | Yes | Yes | Yes |
| Personal characteristics | No | No | Yes | Yes |
| N | 2334 | 2112 | 2111 | 2111 |

*Notes*: Coefficients from an OLS regression. Robust standard errors in parenthesis. * indicates p-value < 0.10; ** p-value < 0.05; *** p-value < 0.01. *Outcome* variable is self-reported answer to the question 'Are you experiencing tensions over the division of work to do in the household at the moment?' on a scale from 0 (no tension at all) to 10 (a lot of tension). *Changed division*: indicator equal to one if the division of the household task is different during the lockdown than before, and zero otherwise (i.e. indicator for the diagonal flows in the Sankey diagrams). *Ch. x fem*: interaction between the indicator for changed division of household labour and female respondent. *Demographic controls*: cubic polynomial in age and indicator for presence of children in the household. *Job status*: controls for respondent and partner's job status, including indicators for working remotely (omitted category); working outside of home (both as essential workers and non-essential workers); work for a family business; government-sponsored training scheme; apprenticeship; employed with other paid work; self-employed; furlough; temporary leave (e.g. maternity leave or ill); student; homemakers; retired. *Personal characteristics*: controls for cooperating with the partner in a Prisoner’s Dilemma game; indicator for risk-seeking behaviours reported in reasons to leave home (see friends, tired of being in the home, getting bored, getting some adrenaline, exercising free will); self-reported life satisfaction; living a worthwhile life; happiness; anxiety; frequency talking with family or friends; indicator for wanting to buy a gift to the partner when lockdown ends. Source: online survey in Italy, UK, USA.

**Supplementary Table 1b: IPW weighted OLS regression predicting tension due to change in allocation of household tasks (all coefficients)**

|  | (1) | (2) | (3) | (4) |
| --- | --- | --- | --- | --- |
|  | Tension over the division of household tasks | | | |
| Changed division: grocery | 0.079 | 0.157 | 0.223 | 0.419 |
|  | (0.195) | (0.176) | (0.165) | (0.276) |
| Ch. grocery x fem |  |  |  | -0.335 |
|  |  |  |  | (0.367) |
| Changed division: clean | 0.714^***^ | 0.660^***^ | 0.597^***^ | 0.495 |
|  | (0.245) | (0.247) | (0.219) | (0.309) |
| Ch. clean x fem |  |  |  | 0.226 |
|  |  |  |  | (0.418) |
| Changed division: cook | -0.331 | -0.547^**^ | -0.393^*^ | -0.522^*^ |
|  | (0.246) | (0.265) | (0.219) | (0.310) |
| Ch. cook x fem |  |  |  | 0.294 |
|  |  |  |  | (0.403) |
| Changed division: gardening | 0.011 | 0.166 | 0.180 | 0.220 |
|  | (0.253) | (0.250) | (0.219) | (0.391) |
| Ch. gardening x fem |  |  |  | -0.099 |
|  |  |  |  | (0.454) |
| Age | 0.002 | 0.001 | 0.005 | 0.005 |
|  | (0.015) | (0.013) | (0.012) | (0.012) |
| Age squared | 0.000 | 0.000 | 0.000 | 0.000 |
|  | (0.001) | (0.001) | (0.001) | (0.001) |
| Age cubed | -0.000^*^ | -0.000 | -0.000^*^ | -0.000^*^ |
|  | (0.000) | (0.000) | (0.000) | (0.000) |
| Children present | 0.900^***^ | 0.984^***^ | 1.069^***^ | 1.064^***^ |
|  | (0.203) | (0.176) | (0.167) | (0.168) |
| Female | 0.513^**^ | 0.436^***^ | 0.200 | 0.228 |
|  | (0.210) | (0.165) | (0.158) | (0.191) |
| UK survey | 0.032 | -0.055 | -0.509^**^ | -0.515^**^ |
|  | (0.199) | (0.195) | (0.202) | (0.202) |
| US survey | -0.053 | 0.027 | -0.365^**^ | -0.368^**^ |
|  | (0.208) | (0.166) | (0.182) | (0.181) |
| Work outside home as essential worker |  | 0.353 | 0.321 | 0.330 |
|  |  | (0.508) | (0.449) | (0.446) |
| In furlough |  | 0.807 | 0.835 | 0.832 |
|  |  | (0.873) | (0.749) | (0.741) |
| Self-employed or freelance |  | -0.450^**^ | -0.451^**^ | -0.450^**^ |
|  |  | (0.211) | (0.203) | (0.200) |
| Working for your own or family business |  | -0.348 | -0.314 | -0.283 |
|  |  | (0.353) | (0.367) | (0.367) |
| Ill, maternity leave, on holiday, or temp leave |  | 0.340 | 0.326 | 0.353 |
|  |  | (0.384) | (0.416) | (0.422) |
| Full time responsibility for family and home |  | -0.184 | -0.159 | -0.137 |
|  |  | (0.250) | (0.233) | (0.234) |
| In education |  | 0.508 | 0.260 | 0.248 |
|  |  | (0.401) | (0.363) | (0.361) |
| Retired |  | -0.403 | -0.286 | -0.267 |
|  |  | (0.343) | (0.339) | (0.330) |
| Partner works outside home as essential worker |  | -0.096 | -0.021 | -0.023 |
|  |  | (0.229) | (0.226) | (0.224) |
| Partner in furlough |  | 0.385 | 0.237 | 0.246 |
|  |  | (0.322) | (0.287) | (0.285) |
| Partner self-employed or freelance |  | 0.377^*^ | 0.425^**^ | 0.427^**^ |
|  |  | (0.206) | (0.194) | (0.192) |
| Partner working for your own or family business |  | 0.640 | 0.419 | 0.460 |
|  |  | (0.561) | (0.687) | (0.679) |
| Partner ill, maternity leave, on holiday, or temp leave |  | 0.431 | 0.550 | 0.579 |
|  |  | (0.578) | (0.527) | (0.528) |
| Partner full time responsible for family and home |  | 0.252 | 0.059 | 0.096 |
|  |  | (0.258) | (0.292) | (0.286) |
| Partner in education |  | 0.874 | 1.125^**^ | 1.152^**^ |
|  |  | (0.661) | (0.509) | (0.503) |
| Partner retired |  | 1.014^*^ | 0.990^*^ | 0.991^*^ |
|  |  | (0.616) | (0.574) | (0.571) |
| Cooperate with Partner in the dilemma game |  |  | 0.133 | 0.127 |
|  |  |  | (0.183) | (0.184) |
| Risk-seeking |  |  | 0.530^**^ | 0.527^**^ |
|  |  |  | (0.226) | (0.227) |
| Life satisfaction |  |  | -0.009 | -0.002 |
|  |  |  | (0.054) | (0.055) |
| Life worthwhile |  |  | -0.099^**^ | -0.101^**^ |
|  |  |  | (0.048) | (0.047) |
| Happy |  |  | -0.155^**^ | -0.160^**^ |
|  |  |  | (0.074) | (0.075) |
| Anxious |  |  | 0.140^***^ | 0.139^***^ |
|  |  |  | (0.031) | (0.031) |
| Frequency talking with friends/family in lockdown |  |  | 0.035 | 0.037 |
|  |  |  | (0.075) | (0.075) |
| Wants to gift partner |  |  | -0.796^***^ | -0.797^***^ |
|  |  |  | (0.227) | (0.226) |
| Constant | 1.412^**^ | 1.272^*^ | 2.396^***^ | 2.394^***^ |
|  | (0.629) | (0.657) | (0.749) | (0.749) |
| Demographic controls | Yes | Yes | Yes | Yes |
| Job status | No | Yes | Yes | Yes |
| Personal characteristics | No | No | Yes | Yes |
| IPW | Yes | Yes | Yes | Yes |
| N | 2334 | 2112 | 2111 | 2111 |

*Notes*: Coefficients from an OLS regression. Robust standard errors in parenthesis. * indicates p-value < 0.10; ** p-value < 0.05; *** p-value < 0.01. *Outcome* variable is self-reported answer to the question 'Are you experiencing tensions over the division of work to do in the household at the moment?' on a scale from 0 (no tension at all) to 10 (a lot of tension). *Changed division*: indicator equal to one if the division of the household task is different during the lockdown than before, and zero otherwise (i.e. indicator for the diagonal flows in the Sankey diagrams). *Ch. x fem*: interaction between the indicator for changed division of household labour and female respondent. *Demographic controls*: cubic polynomial in age and indicator for presence of children in the household. *Job status*: controls for respondent and partner's job status, including indicators for working remotely (omitted category); working outside of home (both as essential workers and non-essential workers); work for a family business; government-sponsored training scheme; apprenticeship; employed with other paid work; self-employed; furlough; temporary leave (e.g. maternity leave or ill); student; homemakers; retired. *Personal characteristics*: controls for cooperating with the partner in a Prisoner’s Dilemma game; indicator for risk-seeking behaviours reported in reasons to leave home (see friends, tired of being in the home, getting bored, getting some adrenaline, exercising free will); self-reported life satisfaction; living a worthwhile life; happiness; anxiety; frequency talking with family or friends; indicator for wanting to buy a gift to the partner when lockdown ends. *IPW*: inverse probability weighting performed to make the (non-representative) Italian sample resemble the other two; IP weights constructed via a logit regression where the outcome variable is an indicator for being part of the Italian sample and the observable characteristics explanatory variables are gender, age, age squared, age cube, presence of children, and several indicator variables for the job status (essential job, furloughed, freelance, family job, on leave, homemaker, in education, retired). The predicted probability p ̂ from this logit regression is used to construct IPW weights: (1-p ̂)/p ̂ for the Italian sample and 1 for the rest. Source: online survey in Italy, UK, USA.

**Supplementary Table 2: OLS regression predicting tension due to change in allocation of household tasks, including childcare (all coefficients)**

|  | (1) | (2) | (3) | (4) |
| --- | --- | --- | --- | --- |
|  | Tension over the division of household tasks | | | |
| Changed division: childcare | 0.471* | 0.358 | 0.361 | 1.017** |
|  | (0.253) | (0.266) | (0.253) | (0.429) |
| Ch. childcare x fem |  |  |  | -1.060** |
|  |  |  |  | (0.524) |
| Changed division: grocery | 0.241 | 0.203 | 0.179 | 0.118 |
|  | (0.205) | (0.208) | (0.205) | (0.448) |
| Ch. grocery x fem |  |  |  | 0.064 |
|  |  |  |  | (0.500) |
| Changed division: clean | 0.227 | 0.398 | 0.283 | 0.621 |
|  | (0.249) | (0.259) | (0.243) | (0.490) |
| Ch. clean x fem |  |  |  | -0.422 |
|  |  |  |  | (0.563) |
| Changed division: cook | -0.101 | -0.255 | -0.212 | -0.291 |
|  | (0.304) | (0.298) | (0.303) | (0.478) |
| Ch. cook x fem |  |  |  | 0.089 |
|  |  |  |  | (0.606) |
| Changed division: gardening | 0.636** | 0.723** | 0.569* | 0.746 |
|  | (0.314) | (0.336) | (0.325) | (0.748) |
| Ch. gardening x fem |  |  |  | -0.261 |
|  |  |  |  | (0.811) |
| Age | 0.015 | 0.014 | -0.007 | -0.008 |
|  | (0.018) | (0.019) | (0.019) | (0.019) |
| Age squared | 0.000 | -0.000 | -0.000 | -0.000 |
|  | (0.001) | (0.001) | (0.001) | (0.001) |
| Age cubed | -0.000* | -0.000** | -0.000 | -0.000 |
|  | (0.000) | (0.000) | (0.000) | (0.000) |
| Female | 0.476** | 0.588** | 0.382 | 0.674** |
|  | (0.219) | (0.235) | (0.235) | (0.275) |
| UK survey | 0.090 | 0.077 | -0.365 | -0.390 |
|  | (0.248) | (0.278) | (0.285) | (0.285) |
| US survey | -0.068 | -0.191 | -0.410 | -0.494* |
|  | (0.253) | (0.270) | (0.260) | (0.261) |
| Work outside home as essential worker |  | 0.203 | 0.155 | 0.133 |
|  |  | (0.316) | (0.314) | (0.314) |
| In furlough |  | 0.565 | 0.470 | 0.434 |
|  |  | (0.543) | (0.472) | (0.483) |
| Self-employed or freelance |  | -0.300 | -0.365 | -0.373 |
|  |  | (0.255) | (0.233) | (0.233) |
| Working for your own or family business |  | 0.194 | 0.292 | 0.277 |
|  |  | (0.731) | (0.650) | (0.661) |
| Ill, maternity leave, on holiday, or temp leave |  | 0.017 | 0.131 | 0.136 |
|  |  | (0.470) | (0.441) | (0.455) |
| Full time responsibility for family and home |  | -0.182 | -0.216 | -0.249 |
|  |  | (0.288) | (0.273) | (0.271) |
| In education |  | 0.742 | 0.607 | 0.630 |
|  |  | (0.979) | (1.071) | (0.971) |
| Retired |  | -0.680 | -0.738* | -0.633 |
|  |  | (0.507) | (0.424) | (0.420) |
| Partner works outside home as essential worker |  | 0.117 | 0.036 | 0.048 |
|  |  | (0.251) | (0.235) | (0.235) |
| Partner in furlough |  | 0.077 | 0.311 | 0.366 |
|  |  | (0.434) | (0.429) | (0.427) |
| Partner self-employed or freelance |  | 0.246 | 0.210 | 0.185 |
|  |  | (0.266) | (0.257) | (0.257) |
| Partner working for your own or family business |  | 0.674 | 0.487 | 0.557 |
|  |  | (0.836) | (0.852) | (0.836) |
| Partner ill, maternity leave, on holiday, or temp leave |  | 0.671 | 0.680 | 0.604 |
|  |  | (0.754) | (0.719) | (0.742) |
| Partner full time responsible for family and home |  | -0.168 | -0.128 | -0.187 |
|  |  | (0.416) | (0.395) | (0.403) |
| Partner in education |  | -0.972 | -1.224 | -0.872 |
|  |  | (1.083) | (1.251) | (1.200) |
| Partner retired |  | 1.090* | 0.902 | 0.776 |
|  |  | (0.616) | (0.663) | (0.615) |
| Cooperate with Partner in the dilemma game |  |  | -0.331* | -0.317 |
|  |  |  | (0.201) | (0.202) |
| Risk-seeking |  |  | 0.407 | 0.383 |
|  |  |  | (0.362) | (0.369) |
| Life satisfaction |  |  | -0.240*** | -0.238*** |
|  |  |  | (0.067) | (0.068) |
| Life worthwhile |  |  | -0.011 | -0.019 |
|  |  |  | (0.063) | (0.063) |
| Happy |  |  | -0.056 | -0.057 |
|  |  |  | (0.067) | (0.067) |
| Anxious |  |  | 0.156*** | 0.156*** |
|  |  |  | (0.038) | (0.038) |
| Frequency talking with friends/family in lockdown |  |  | -0.107 | -0.106 |
|  |  |  | (0.080) | (0.081) |
| Wants to gift partner |  |  | -0.013 | 0.030 |
|  |  |  | (0.309) | (0.311) |
| Demographic controls | Yes | Yes | Yes | Yes |
| Job status | No | Yes | Yes | Yes |
| Personal characteristics | No | No | Yes | Yes |
| N | 840 | 795 | 795 | 795 |
|  |  |  |  |  |
|  |  |  |  |  |

*Notes*: Coefficients from an OLS regression. Robust standard errors in parenthesis. * indicates p-value < 0.10; ** p-value < 0.05; *** p-value < 0.01. *Outcome* variable is self-reported answer to the question 'Are you experiencing tensions over the division of work to do in the household at the moment?' on a scale from 0 (no tension at all) to 10 (a lot of tension). *Changed division*: indicator equal to one if the division of the household task is different during the lockdown than before, and zero otherwise (i.e. indicator for the diagonal flows in the Sankey diagrams). *Ch. x fem*: interaction between the indicator for changed division of household labour and female respondent. *Demographic controls*: cubic polynomial in age and indicator for presence of children in the household. *Job status*: controls for respondent and partner's job status, including indicators for working remotely (omitted category); working outside of home (both as essential workers and non-essential workers); work for a family business; government-sponsored training scheme; apprenticeship; employed with other paid work; self-employed; furlough; temporary leave (e.g. maternity leave or ill); student; homemakers; retired. *Personal characteristics*: controls for cooperating with the partner in a Prisoner’s Dilemma game; indicator for risk-seeking behaviours reported in reasons to leave home (see friends, tired of being in the home, getting bored, getting some adrenaline, exercising free will); self-reported life satisfaction; living a worthwhile life; happiness; anxiety; frequency talking with family or friends; indicator for wanting to buy a gift to the partner when lockdown ends. Source: online survey in Italy, UK, USA.

**Supplementary Table 3a: OLS regression predicting increased quarrelling during the lockdown, using change in in allocation of household tasks (all coefficients)**

|  | (1) | (2) | (3) | (4) |
| --- | --- | --- | --- | --- |
|  | More quarrelling during the lockdown | | | |
|  |  |  |  |  |
| Changed division: grocery | 0.118* | 0.041 | 0.053 | -0.120 |
|  | (0.071) | (0.075) | (0.077) | (0.162) |
| Ch. grocery x fem |  |  |  | 0.217 |
|  |  |  |  | (0.185) |
| Changed division: clean | 0.160* | 0.171* | 0.147 | 0.085 |
|  | (0.093) | (0.098) | (0.099) | (0.175) |
| Ch. clean x fem |  |  |  | 0.102 |
|  |  |  |  | (0.209) |
| Changed division: cook | 0.176* | 0.122 | 0.164 | 0.464*** |
|  | (0.103) | (0.110) | (0.113) | (0.179) |
| Ch. cook x fem |  |  |  | -0.486** |
|  |  |  |  | (0.232) |
| Changed division: gardening | 0.169 | 0.215* | 0.200* | 0.361 |
|  | (0.106) | (0.112) | (0.113) | (0.231) |
| Ch. gardening x fem |  |  |  | -0.205 |
|  |  |  |  | (0.265) |
| Age | -0.014*** | -0.014*** | -0.012** | -0.013** |
|  | (0.005) | (0.005) | (0.005) | (0.005) |
| Age squared | 0.000 | 0.000* | 0.000* | 0.000* |
|  | (0.000) | (0.000) | (0.000) | (0.000) |
| Age cubed | 0.000 | 0.000 | 0.000 | 0.000 |
|  | (0.000) | (0.000) | (0.000) | (0.000) |
| Children present | 0.192*** | 0.149* | 0.206** | 0.192** |
|  | (0.074) | (0.080) | (0.082) | (0.082) |
| Female | 0.146** | 0.173** | 0.122 | 0.142 |
|  | (0.072) | (0.080) | (0.083) | (0.102) |
| UK survey | 0.234*** | 0.266*** | 0.073 | 0.069 |
|  | (0.080) | (0.089) | (0.098) | (0.098) |
| US survey | 0.047 | 0.044 | -0.161 | -0.156 |
|  | (0.088) | (0.097) | (0.109) | (0.109) |
| Work outside home as essential worker |  | -0.040 | -0.034 | -0.026 |
|  |  | (0.115) | (0.117) | (0.116) |
| In furlough |  | -0.120 | -0.181 | -0.181 |
|  |  | (0.151) | (0.153) | (0.153) |
| Self-employed or freelance |  | -0.112 | -0.148 | -0.142 |
|  |  | (0.096) | (0.098) | (0.098) |
| Working for your own or family business |  | 0.057 | 0.089 | 0.070 |
|  |  | (0.221) | (0.224) | (0.226) |
| Ill, maternity leave, on holiday, or temp leave |  | 0.166 | 0.122 | 0.119 |
|  |  | (0.189) | (0.197) | (0.198) |
| Full time responsibility for family and home |  | 0.088 | 0.076 | 0.073 |
|  |  | (0.127) | (0.129) | (0.129) |
| In education |  | 0.076 | 0.055 | 0.055 |
|  |  | (0.179) | (0.183) | (0.183) |
| Retired |  | -0.160 | -0.164 | -0.151 |
|  |  | (0.178) | (0.182) | (0.183) |
| Partner works outside home as essential worker |  | -0.064 | -0.057 | -0.057 |
|  |  | (0.097) | (0.099) | (0.100) |
| Partner in furlough |  | 0.111 | 0.106 | 0.107 |
|  |  | (0.142) | (0.146) | (0.146) |
| Partner self-employed or freelance |  | -0.012 | -0.033 | -0.035 |
|  |  | (0.101) | (0.102) | (0.103) |
| Partner working for your own or family business |  | -0.089 | -0.082 | -0.093 |
|  |  | (0.282) | (0.288) | (0.283) |
| Partner ill, maternity leave, on holiday, or temp leave |  | 0.301 | 0.329 | 0.331 |
|  |  | (0.245) | (0.249) | (0.252) |
| Partner full time responsible for family and home |  | 0.353** | 0.337** | 0.351** |
|  |  | (0.158) | (0.158) | (0.159) |
| Partner in education |  | -0.028 | 0.001 | 0.024 |
|  |  | (0.222) | (0.220) | (0.220) |
| Partner retired |  | -0.150 | -0.144 | -0.149 |
|  |  | (0.154) | (0.155) | (0.156) |
| Cooperate with Partner in the dilemma game |  |  | 0.028 | 0.032 |
|  |  |  | (0.076) | (0.077) |
| Risk-seeking |  |  | 0.235** | 0.237** |
|  |  |  | (0.117) | (0.117) |
| Life satisfaction |  |  | -0.011 | -0.014 |
|  |  |  | (0.023) | (0.023) |
| Life worthwhile |  |  | -0.026 | -0.027 |
|  |  |  | (0.021) | (0.021) |
| Happy |  |  | -0.082*** | -0.080*** |
|  |  |  | (0.022) | (0.022) |
| Anxious |  |  | 0.023* | 0.024* |
|  |  |  | (0.013) | (0.013) |
| Frequency talking with friends/family in lockdown |  |  | -0.026 | -0.026 |
|  |  |  | (0.031) | (0.032) |
| Wants to gift partner |  |  | -0.465*** | -0.474*** |
|  |  |  | (0.150) | (0.149) |
| Demographic controls | Yes | Yes | Yes | Yes |
| Job status | No | Yes | Yes | Yes |
| Personal characteristics | No | No | Yes | Yes |
| N | 2297 | 2079 | 2079 | 2079 |

*Notes*: Coefficients from an OLS regression. Robust standard errors in parenthesis. * indicates p-value < 0.10; ** p-value < 0.05; *** p-value < 0.01. *Outcome* variable is an indicator equal to one if the respondent reported a higher frequency of quarrelling during vs before the lockdown (constructed as the first difference of two questions ‘How often do you and your partner/flatmate quarrel since isolation/usually, before the lockdown?' on a scale from 1 (never) to 6 (all of the time)). *Changed division*: indicator equal to one if the division of the household task is different during the lockdown than before, and zero otherwise (i.e. indicator for the diagonal flows in the Sankey diagrams). *Ch. x fem*: interaction between the indicator for changed division of household labour and female respondent. *Demographic controls*: cubic polynomial in age and indicator for presence of children in the household. *Job status*: controls for respondent and partner's job status, including indicators for working remotely (omitted category); working outside of home (both as essential workers and non-essential workers); work for a family business; government-sponsored training scheme; apprenticeship; employed with other paid work; self-employed; furlough; temporary leave (e.g. maternity leave or ill); student; homemakers; retired. *Personal characteristics*: controls for cooperating with the partner in a Prisoner’s Dilemma game; indicator for risk-seeking behaviours reported in reasons to leave home (see friends, tired of being in the home, getting bored, getting some adrenaline, exercising free will); self-reported life satisfaction; living a worthwhile life; happiness; anxiety; frequency talking with family or friends; indicator for wanting to buy a gift to the partner when lockdown ends. Source: online survey in Italy, UK, USA.

**Supplementary Table 3b: IPW weighted OLS regression predicting increased quarrelling during the lockdown, using change in in allocation of household tasks (all coefficients)**

|  | (1) | (2) | (3) | (4) |
| --- | --- | --- | --- | --- |
|  | More quarrelling during the lockdown | | | |
| Changed division: grocery | 0.100 | 0.011 | 0.047 | -0.128 |
|  | (0.087) | (0.085) | (0.085) | (0.157) |
| Ch. grocery x fem |  |  |  | 0.250 |
|  |  |  |  | (0.189) |
| Changed division: clean | 0.319^**^ | 0.317^**^ | 0.257^**^ | 0.124 |
|  | (0.132) | (0.130) | (0.121) | (0.193) |
| Ch. clean x fem |  |  |  | 0.237 |
|  |  |  |  | (0.240) |
| Changed division: cook | 0.099 | 0.048 | 0.133 | 0.345^*^ |
|  | (0.121) | (0.131) | (0.128) | (0.190) |
| Ch. cook x fem |  |  |  | -0.385 |
|  |  |  |  | (0.251) |
| Changed division: gardening | 0.183 | 0.234^*^ | 0.262^**^ | 0.220 |
|  | (0.131) | (0.134) | (0.129) | (0.232) |
| Ch. gardening x fem |  |  |  | 0.068 |
|  |  |  |  | (0.279) |
| Age | -0.013^**^ | -0.015^**^ | -0.014^**^ | -0.015^**^ |
|  | (0.006) | (0.007) | (0.006) | (0.006) |
| Age squared | 0.000 | 0.000 | 0.000 | 0.000 |
|  | (0.000) | (0.000) | (0.000) | (0.000) |
| Age cubed | -0.000 | -0.000 | -0.000 | -0.000 |
|  | (0.000) | (0.000) | (0.000) | (0.000) |
| Children present | 0.221^**^ | 0.192^*^ | 0.250^***^ | 0.240^**^ |
|  | (0.089) | (0.099) | (0.096) | (0.096) |
| Female | 0.338^***^ | 0.392^***^ | 0.303^***^ | 0.245^**^ |
|  | (0.090) | (0.093) | (0.093) | (0.115) |
| UK survey | 0.405^***^ | 0.445^***^ | 0.193^*^ | 0.201^*^ |
|  | (0.092) | (0.099) | (0.108) | (0.107) |
| US survey | 0.200^**^ | 0.218^**^ | -0.049 | -0.031 |
|  | (0.102) | (0.105) | (0.120) | (0.120) |
| Work outside home as essential worker |  | -0.224^*^ | -0.205^*^ | -0.216^*^ |
|  |  | (0.119) | (0.120) | (0.121) |
| In furlough |  | -0.381^**^ | -0.456^***^ | -0.439^***^ |
|  |  | (0.174) | (0.161) | (0.158) |
| Self-employed or freelance |  | -0.218^**^ | -0.253^**^ | -0.261^**^ |
|  |  | (0.107) | (0.110) | (0.112) |
| Working for your own or family business |  | -0.068 | -0.078 | -0.067 |
|  |  | (0.240) | (0.264) | (0.264) |
| Ill, maternity leave, on holiday, or temp leave |  | -0.055 | -0.104 | -0.110 |
|  |  | (0.209) | (0.224) | (0.225) |
| Full time responsibility for family and home |  | 0.089 | 0.060 | 0.057 |
|  |  | (0.149) | (0.143) | (0.142) |
| In education |  | 0.084 | -0.042 | -0.028 |
|  |  | (0.196) | (0.189) | (0.187) |
| Retired |  | 0.041 | 0.072 | 0.072 |
|  |  | (0.193) | (0.197) | (0.199) |
| Partner works outside home as essential worker |  | -0.112 | -0.081 | -0.072 |
|  |  | (0.111) | (0.112) | (0.112) |
| Partner in furlough |  | 0.295 | 0.253 | 0.259 |
|  |  | (0.182) | (0.165) | (0.165) |
| Partner self-employed or freelance |  | 0.040 | 0.023 | 0.030 |
|  |  | (0.122) | (0.122) | (0.123) |
| Partner working for your own or family business |  | -0.138 | -0.129 | -0.129 |
|  |  | (0.313) | (0.368) | (0.362) |
| Partner ill, maternity leave, on holiday, or temp leave |  | 0.270 | 0.366 | 0.359 |
|  |  | (0.261) | (0.278) | (0.280) |
| Partner full time responsible for family and home |  | 0.511^***^ | 0.462^**^ | 0.436^**^ |
|  |  | (0.195) | (0.188) | (0.187) |
| Partner in education |  | 0.232 | 0.303 | 0.303 |
|  |  | (0.329) | (0.290) | (0.284) |
| Partner retired |  | -0.169 | -0.177 | -0.168 |
|  |  | (0.173) | (0.171) | (0.172) |
| Cooperate with Partner in the dilemma game |  |  | 0.016 | 0.022 |
|  |  |  | (0.087) | (0.088) |
| Risk-seeking |  |  | 0.248^*^ | 0.251^*^ |
|  |  |  | (0.139) | (0.138) |
| Life satisfaction |  |  | -0.016 | -0.020 |
|  |  |  | (0.025) | (0.025) |
| Life worthwhile |  |  | -0.037 | -0.035 |
|  |  |  | (0.024) | (0.024) |
| Happy |  |  | -0.097^***^ | -0.095^***^ |
|  |  |  | (0.025) | (0.024) |
| Anxious |  |  | 0.035^**^ | 0.035^**^ |
|  |  |  | (0.015) | (0.015) |
| Frequency talking with friends/family in lockdown |  |  | -0.052 | -0.053 |
|  |  |  | (0.035) | (0.035) |
| Wants to gift partner |  |  | -0.581^***^ | -0.587^***^ |
|  |  |  | (0.182) | (0.184) |
| Constant | -1.169^***^ | -1.029^***^ | 0.175 | 0.228 |
|  | (0.351) | (0.362) | (0.398) | (0.399) |
| Demographic controls | Yes | Yes | Yes | Yes |
| Job status | No | Yes | Yes | Yes |
| Personal characteristics | No | No | Yes | Yes |
| IPW | Yes | Yes | Yes | Yes |
| N | 2297 | 2079 | 2079 | 2079 |

*Notes*: Coefficients from an OLS regression. Robust standard errors in parenthesis. * indicates p-value < 0.10; ** p-value < 0.05; *** p-value < 0.01. *Outcome* variable is an indicator equal to one if the respondent reported a higher frequency of quarrelling during vs before the lockdown (constructed as the first difference of two questions ‘How often do you and your partner/flatmate quarrel since isolation/usually, before the lockdown?' on a scale from 1 (never) to 6 (all of the time)). *Changed division*: indicator equal to one if the division of the household task is different during the lockdown than before, and zero otherwise (i.e. indicator for the diagonal flows in the Sankey diagrams). *Ch. x fem*: interaction between the indicator for changed division of household labour and female respondent. *Demographic controls*: cubic polynomial in age and indicator for presence of children in the household. *Job status*: controls for respondent and partner's job status, including indicators for working remotely (omitted category); working outside of home (both as essential workers and non-essential workers); work for a family business; government-sponsored training scheme; apprenticeship; employed with other paid work; self-employed; furlough; temporary leave (e.g. maternity leave or ill); student; homemakers; retired. *Personal characteristics*: controls for cooperating with the partner in a Prisoner’s Dilemma game; indicator for risk-seeking behaviours reported in reasons to leave home (see friends, tired of being in the home, getting bored, getting some adrenaline, exercising free will); self-reported life satisfaction; living a worthwhile life; happiness; anxiety; frequency talking with family or friends; indicator for wanting to buy a gift to the partner when lockdown ends. *IPW*: inverse probability weighting performed to make the (non-representative) Italian sample resemble the other two; IP weights constructed via a logit regression where the outcome variable is an indicator for being part of the Italian sample and the observable characteristics explanatory variables are gender, age, age squared, age cube, presence of children, and several indicator variables for the job status (essential job, furloughed, freelance, family job, on leave, homemaker, in education, retired). The predicted probability p ̂ from this logit regression is used to construct IPW weights: (1-p ̂)/p ̂ for the Italian sample and 1 for the rest. Source: online survey in Italy, UK, USA.

**Supplementary Table 4: OLS regression predicting increased quarrelling during the lockdown, using change in in allocation of household tasks including childcare (all coefficients)**

|  | (1) | (2) | (3) | (4) |
| --- | --- | --- | --- | --- |
|  | More quarrelling during the lockdown | | | |
|  |  |  |  |  |
| Changed division: childcare | 0.256* | 0.291** | 0.296** | 0.389 |
|  | (0.134) | (0.142) | (0.144) | (0.243) |
| Ch. childcare x fem |  |  |  | -0.167 |
|  |  |  |  | (0.300) |
| Changed division: grocery | 0.021 | -0.038 | -0.006 | -0.408 |
|  | (0.110) | (0.115) | (0.118) | (0.309) |
| Ch. grocery x fem |  |  |  | 0.435 |
|  |  |  |  | (0.335) |
| Changed division: clean | 0.063 | 0.088 | 0.060 | -0.239 |
|  | (0.135) | (0.142) | (0.143) | (0.281) |
| Ch. clean x fem |  |  |  | 0.390 |
|  |  |  |  | (0.323) |
| Changed division: cook | 0.076 | 0.020 | 0.051 | 0.641** |
|  | (0.156) | (0.171) | (0.177) | (0.295) |
| Ch. cook x fem |  |  |  | -0.922** |
|  |  |  |  | (0.382) |
| Changed division: gardening | 0.132 | 0.144 | 0.093 | 0.631 |
|  | (0.168) | (0.176) | (0.180) | (0.418) |
| Ch. gardening x fem |  |  |  | -0.680 |
|  |  |  |  | (0.461) |
| Age | -0.013 | -0.012 | -0.019 | -0.017 |
|  | (0.011) | (0.013) | (0.014) | (0.014) |
| Age squared | -0.000 | -0.001 | -0.001 | -0.001 |
|  | (0.001) | (0.001) | (0.001) | (0.001) |
| Age cubed | -0.000 | -0.000 | -0.000 | -0.000 |
|  | (0.000) | (0.000) | (0.000) | (0.000) |
| Female | 0.087 | 0.128 | 0.052 | 0.172 |
|  | (0.123) | (0.143) | (0.152) | (0.200) |
| UK survey | 0.082 | 0.150 | 0.015 | 0.009 |
|  | (0.137) | (0.155) | (0.181) | (0.182) |
| US survey | -0.029 | -0.097 | -0.219 | -0.229 |
|  | (0.142) | (0.163) | (0.181) | (0.182) |
| Work outside home as essential worker |  | 0.033 | 0.010 | 0.037 |
|  |  | (0.176) | (0.183) | (0.183) |
| In furlough |  | -0.155 | -0.272 | -0.305 |
|  |  | (0.291) | (0.282) | (0.285) |
| Self-employed or freelance |  | -0.084 | -0.141 | -0.130 |
|  |  | (0.150) | (0.151) | (0.150) |
| Working for your own or family business |  | 0.195 | 0.272 | 0.248 |
|  |  | (0.361) | (0.356) | (0.364) |
| Ill, maternity leave, on holiday, or temp leave |  | 0.325 | 0.324 | 0.354 |
|  |  | (0.245) | (0.247) | (0.250) |
| Full time responsibility for family and home |  | 0.183 | 0.156 | 0.156 |
|  |  | (0.167) | (0.171) | (0.173) |
| In education |  | 0.885* | 0.853 | 0.863 |
|  |  | (0.529) | (0.555) | (0.566) |
| Retired |  | 0.284 | 0.295 | 0.288 |
|  |  | (0.626) | (0.580) | (0.609) |
| Partner works outside home as essential worker |  | -0.156 | -0.185 | -0.172 |
|  |  | (0.139) | (0.145) | (0.147) |
| Partner in furlough |  | -0.197 | -0.092 | -0.085 |
|  |  | (0.264) | (0.282) | (0.287) |
| Partner self-employed or freelance |  | -0.023 | -0.036 | -0.034 |
|  |  | (0.148) | (0.149) | (0.148) |
| Partner working for your own or family business |  | -0.659 | -0.802 | -0.814 |
|  |  | (0.552) | (0.567) | (0.572) |
| Partner ill, maternity leave, on holiday, or temp leave |  | 0.568 | 0.668* | 0.706** |
|  |  | (0.346) | (0.344) | (0.358) |
| Partner full time responsible for family and home |  | 0.177 | 0.215 | 0.272 |
|  |  | (0.236) | (0.245) | (0.247) |
| Cooperate with Partner in the dilemma game |  |  | -0.193 | -0.199 |
|  |  |  | (0.121) | (0.123) |
| Risk-seeking |  |  | 0.472** | 0.491** |
|  |  |  | (0.208) | (0.211) |
| Life satisfaction |  |  | -0.060* | -0.063* |
|  |  |  | (0.036) | (0.036) |
| Life worthwhile |  |  | 0.015 | 0.015 |
|  |  |  | (0.034) | (0.034) |
| Happy |  |  | -0.083** | -0.082** |
|  |  |  | (0.037) | (0.038) |
| Anxious |  |  | 0.018 | 0.017 |
|  |  |  | (0.022) | (0.022) |
| Frequency talking with friends/family in lockdown |  |  | 0.029 | 0.033 |
|  |  |  | (0.054) | (0.054) |
| Wants to gift partner |  |  | -0.317 | -0.313 |
|  |  |  | (0.209) | (0.207) |
| Demographic controls | Yes | Yes | Yes | Yes |
| Job status | No | Yes | Yes | Yes |
| Personal characteristics | No | No | Yes | Yes |
| N | 836 | 781 | 781 | 778 |

*Notes*: Coefficients from an OLS regression. Robust standard errors in parenthesis. * indicates p-value < 0.10; ** p-value < 0.05; *** p-value < 0.01. *Outcome* variable is an indicator equal to one if the respondent reported a higher frequency of quarrelling during vs before the lockdown (constructed as the first difference of two questions ‘How often do you and your partner/flatmate quarrel since isolation/usually, before the lockdown?' on a scale from 1 (never) to 6 (all of the time). *Changed division*: indicator equal to one if the division of the household task is different during the lockdown than before, and zero otherwise (i.e. indicator for the diagonal flows in the Sankey diagrams). *Ch. x fem*: interaction between the indicator for changed division of household labor and female respondent. *Demographic controls*: cubic polynomial in age and indicator for presence of children in the household. *Job status*: controls for respondent and partner's job status, including indicators for working remotely (omitted category); working outside of home (both as essential workers and non-essential workers); work for a family business; government-sponsored training scheme; apprenticeship; employed with other paid work; self-employed; furlough; temporary leave (e.g. maternity leave or ill); student; homemakers; retired. *Personal characteristics*: controls for cooperating with the partner in a Prisoner’s Dilemma game; indicator for risk-seeking behaviours reported in reasons to leave home (see friends, tired of being in the home, getting bored, getting some adrenaline, exercising free will); self-reported life satisfaction; living a worthwhile life; happiness; anxiety; frequency talking with family or friends; indicator for wanting to buy a gift to the partner when lockdown ends. Source: online survey in Italy, UK, USA.

## Supplementary Figures

**Supplementary Figure S1: Sankey diagrams for the reallocation of cleaning, cooking, laundry, and gardening from before to during the lockdown.**

| Cleaning |  |  |
| --- | --- | --- |
|  | Respondent has relatively more time at home | Respondent has relatively the same time at home |
| Women | 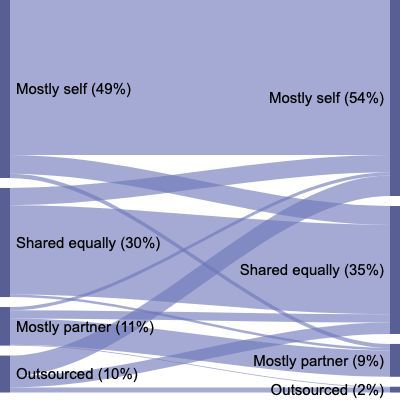 | 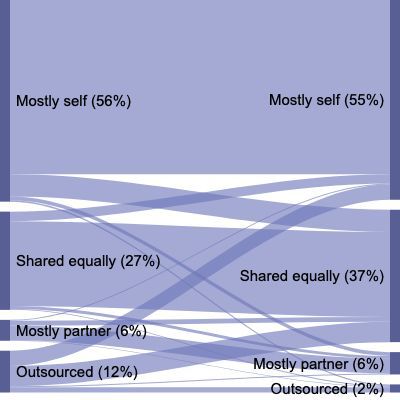 |
| Men | 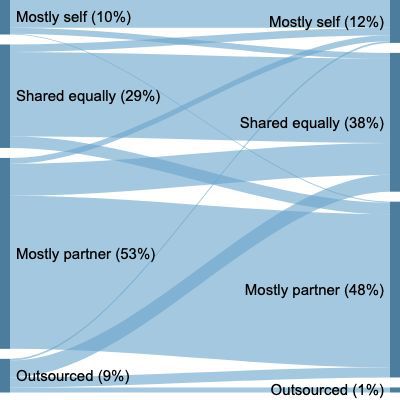 | 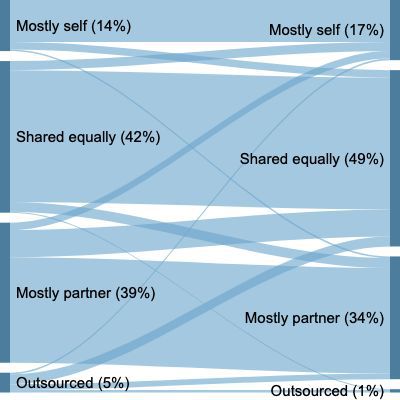 |

| Cooking |  |  |
| --- | --- | --- |
|  | Respondent has relatively more time at home | Respondent has relatively less or the same time at home |
| Women | 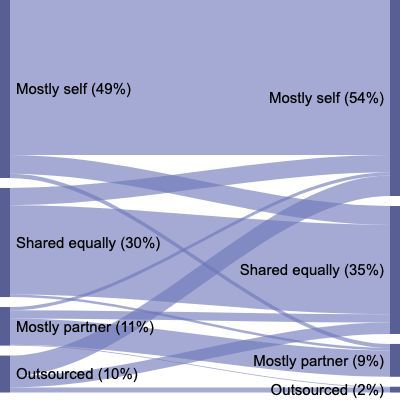 | 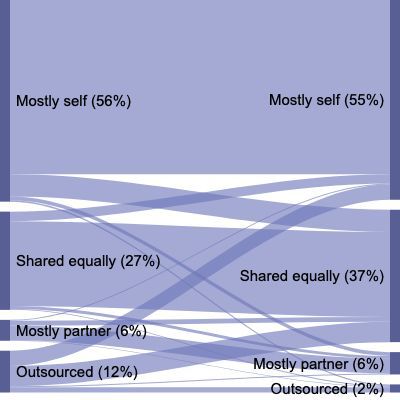 |
| Men | 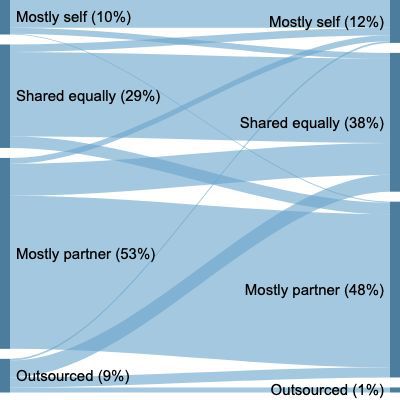 | 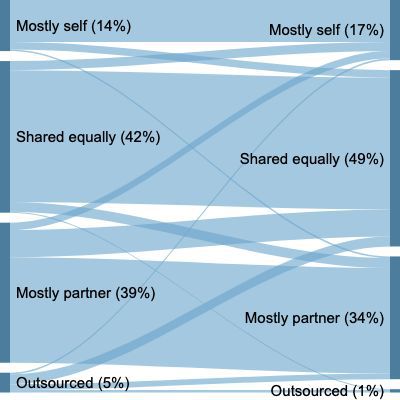 |

| Laundry |  |  |
| --- | --- | --- |
|  | Respondent has relatively more time at home | Respondent has relatively the same time at home |
| Women | 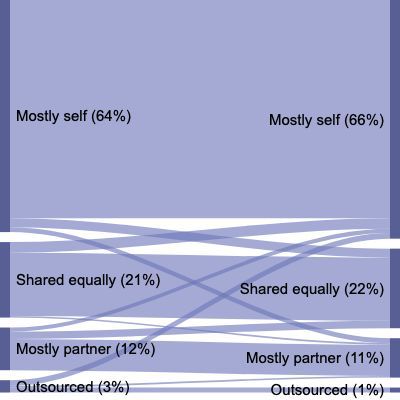 | 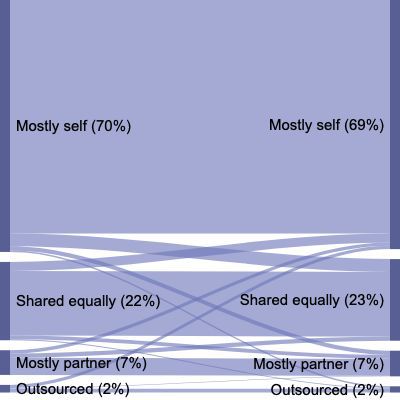 |
| Men | 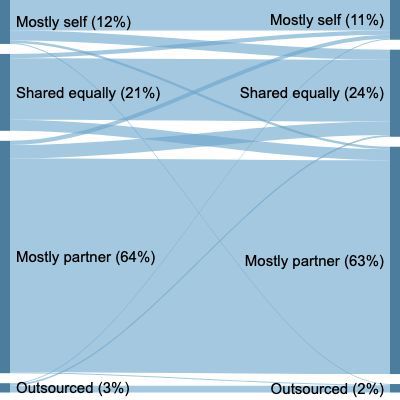 | 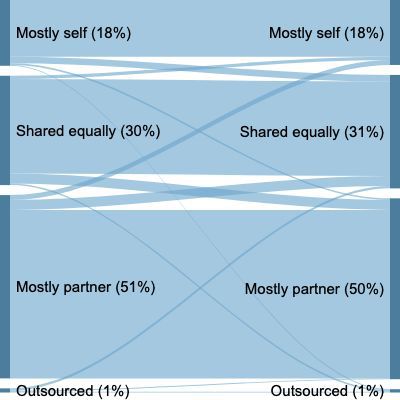 |

| Gardening |  |  |
| --- | --- | --- |
|  | Respondent has relatively more time at home | Respondent has relatively less or the same time at home |
| Women | 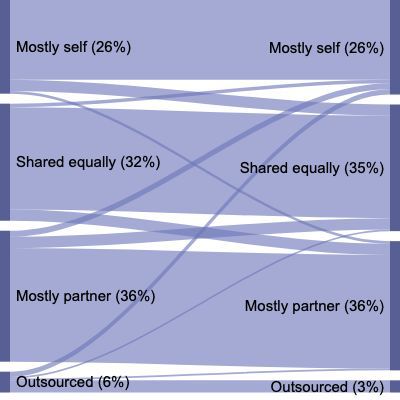 | 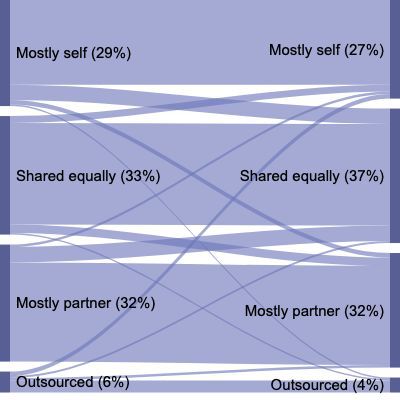 |
| Men | 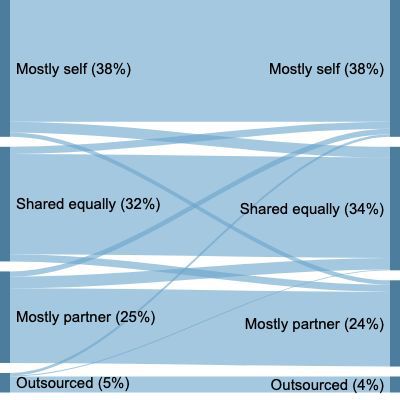 | 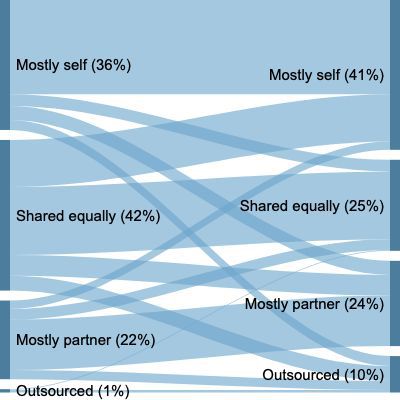 |

*Notes:* The above Sankey diagrams report changes in cleaning, cooking, laundry, and gardening allocation from before the lockdown (left-hand side of each diagram) to during the lockdown (right-hand side of each diagram) for women and men respectively. The figures are split according to whether the respondent has more (left-hand side panel) or the same time (right-hand side panel) at home during lockdown than before relative to their partner. Responses for which the respondent has less time relatively are flipped so that the response is used from the perspective of the respondent’s partner. Source: online survey in Italy, UK, USA. For cleaning, N = 1,296 (women) and 871 (men). For cooking, N = 1,207 (women) and 872 (men). For laundry, N = 1,207 (women) and 871 (men). For gardening, N = 1,157 (women) and 826 (men).

**Supplementary Figure S2: Coefficient plot from ordered probit regressions predicting changes in family chore allocations; replication of Table 1 and also with inverse probability weighting (IPW) of the Italian respondents**

**
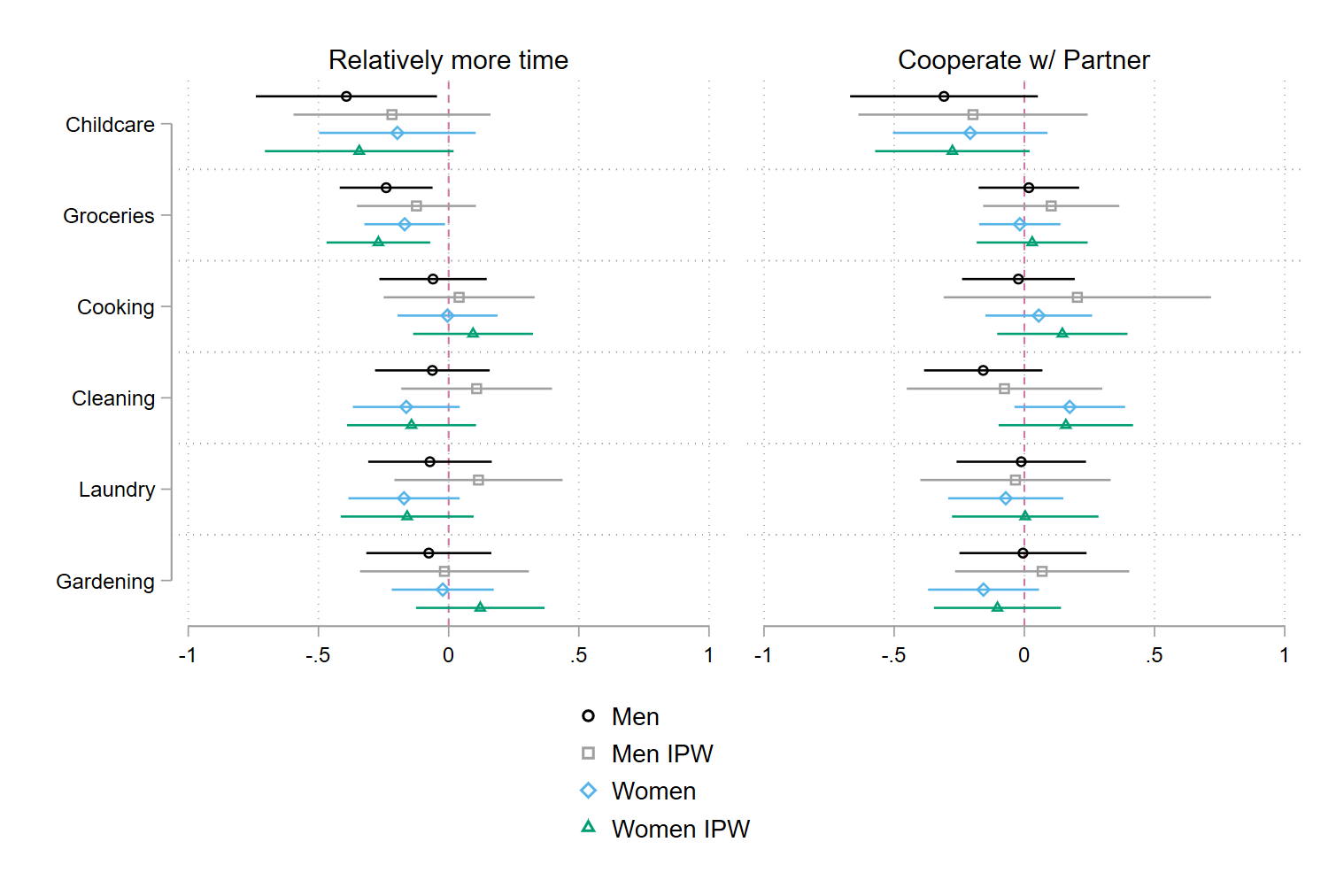
**

**Supplementary Figure S3: Changes in division of cooking from before to during the lockdown, coloured by share of household reporting high tension**

**US**

**UK**

**ITA**


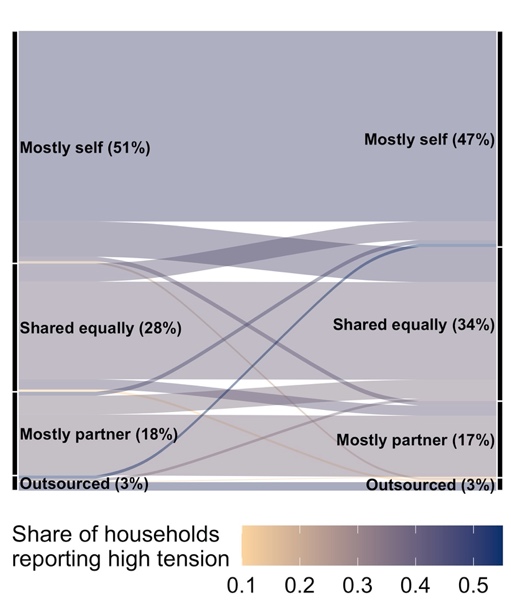

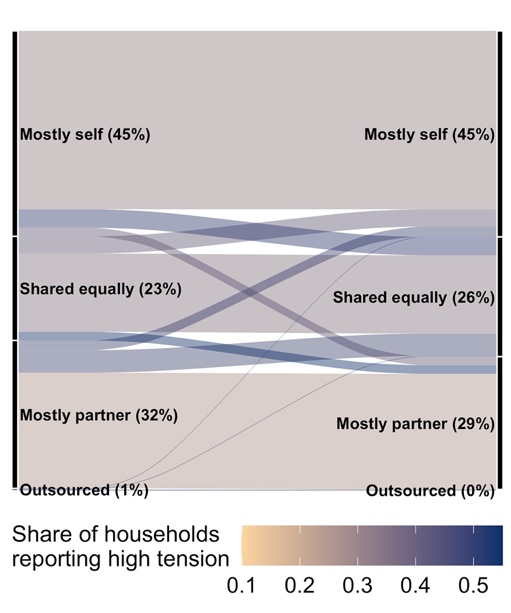

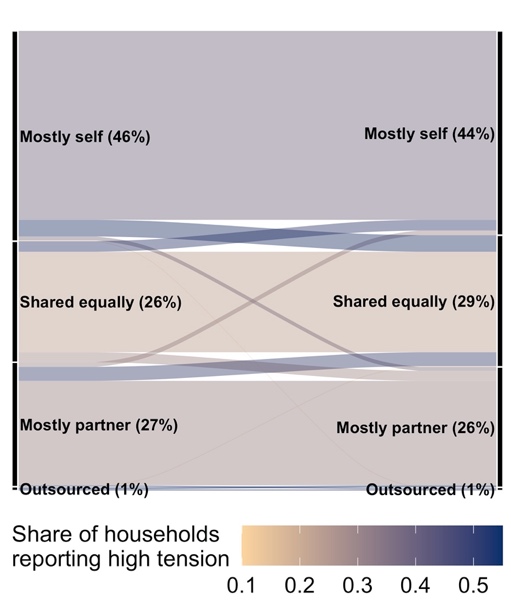


*Notes:* The above Sankey diagrams report changes in cooking allocation from before the lockdown (left-hand side of each diagram) to during the lockdown (right-hand side of each diagram) for each of the countries surveyed. Diagram flows are coloured by the share of respondents reporting high household tensions specifically related to the allocations of household tasks. Darker lines correspond to subsets with higher reported household tensions, and are useful in capturing the effect of task reallocation in lockdown. . Source: online survey in Italy, UK, USA. N = 2,524.

**
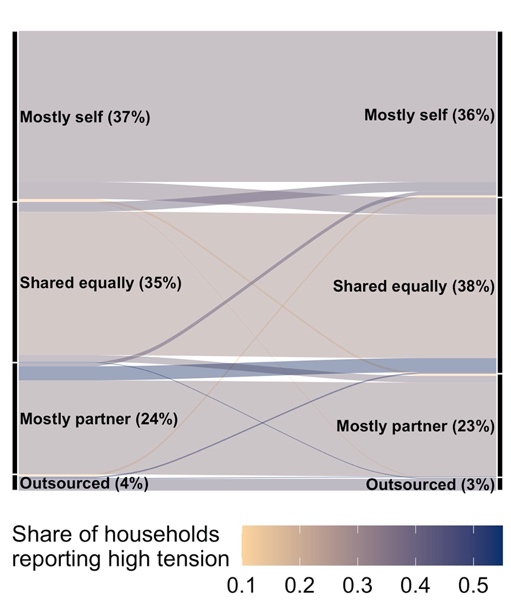

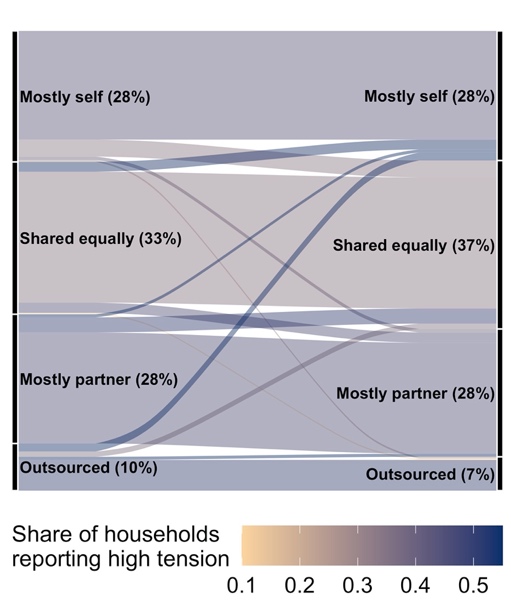

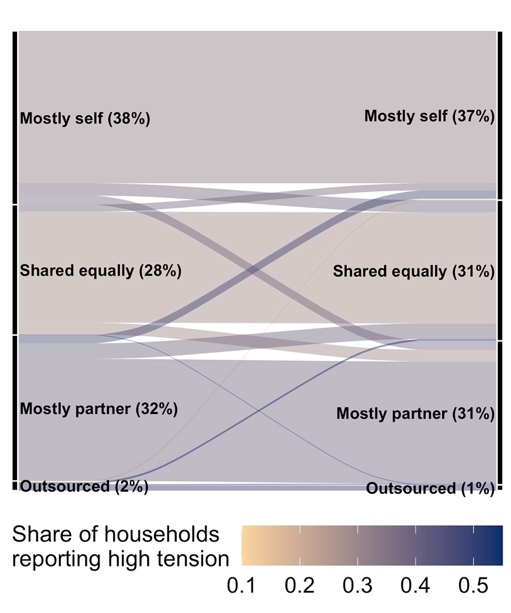
Supplementary Figure S4: Changes in division of gardening from before to during the lockdown, coloured by share of household reporting high tension**

**US**

**UK**

**ITA**

*Notes:* The above Sankey diagrams report changes in gardening allocation from before the lockdown (left-hand side of each diagram) to during the lockdown (right-hand side of each diagram) for each of the countries surveyed. Diagram flows are coloured by the share of respondents reporting high household tensions specifically related to the allocations of household tasks. Darker lines correspond to subsets with higher reported household tensions, and are useful in capturing the effect of task reallocation in lockdown. Source: online survey in Italy, UK, USA. N = 2,367.


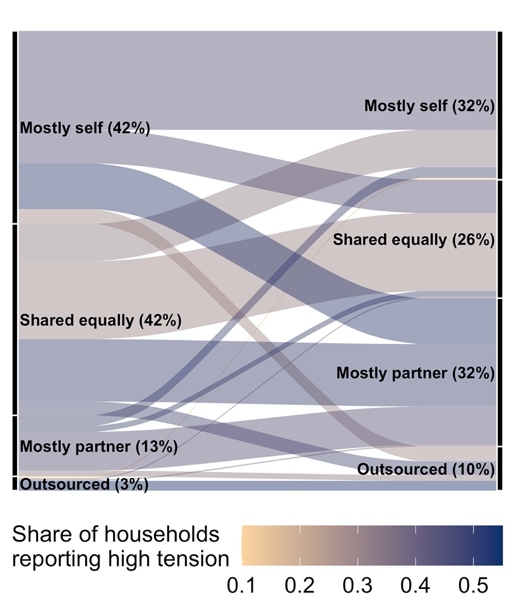

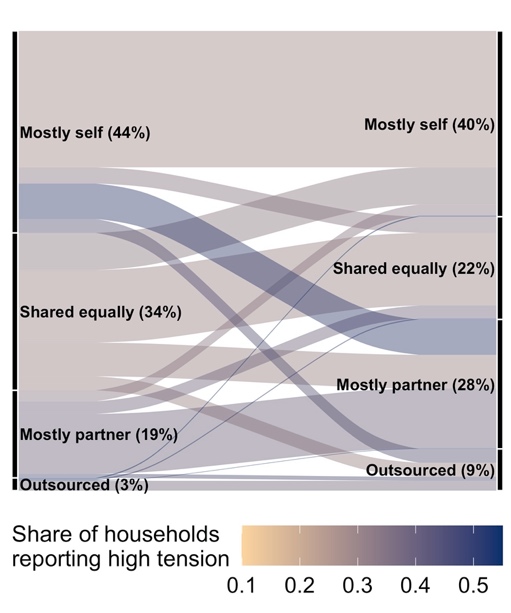

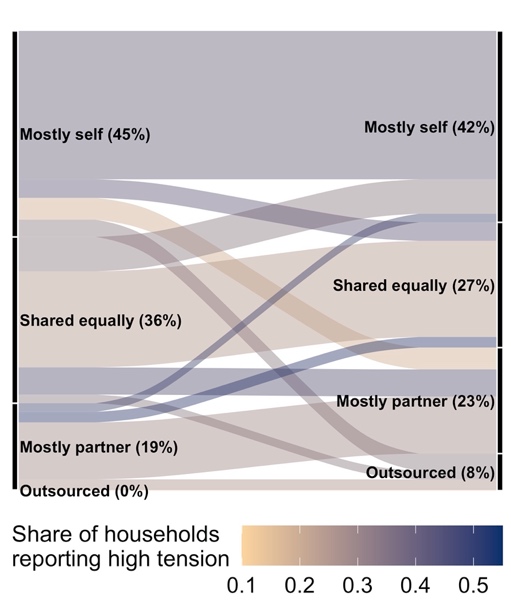
**Supplementary Figure S5: Changes in division of groceries from before to during the lockdown, coloured by share of household reporting high tension**

**US**

**UK**

**ITA**

*Notes:* The above Sankey diagrams report changes in grocery shopping allocation from before the lockdown (left-hand side of each diagram) to during the lockdown (right-hand side of each diagram) for each of the countries surveyed. Diagram flows are coloured by the share of respondents reporting high household tensions specifically related to the allocations of household tasks. Darker lines correspond to subsets with higher reported household tensions, and are useful in capturing the effect of task reallocation in lockdown. Source: online survey in Italy, UK, USA. N = 2,520.


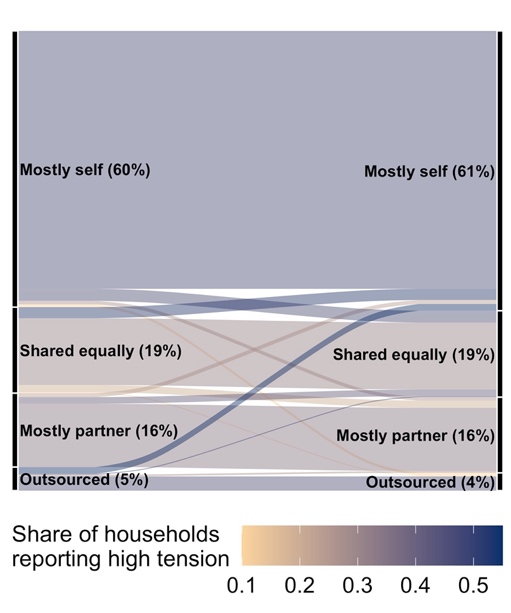

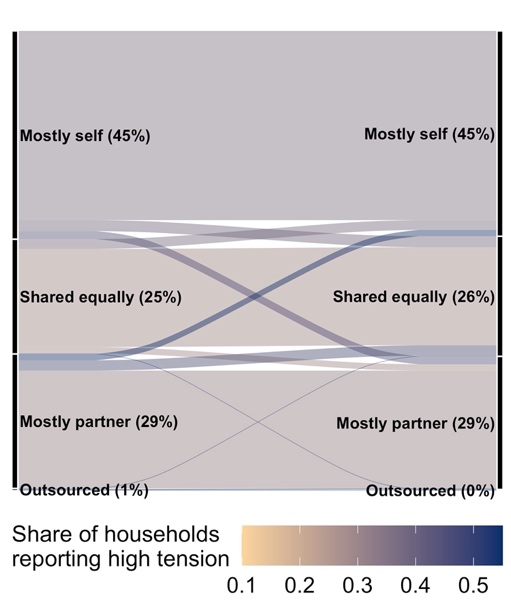

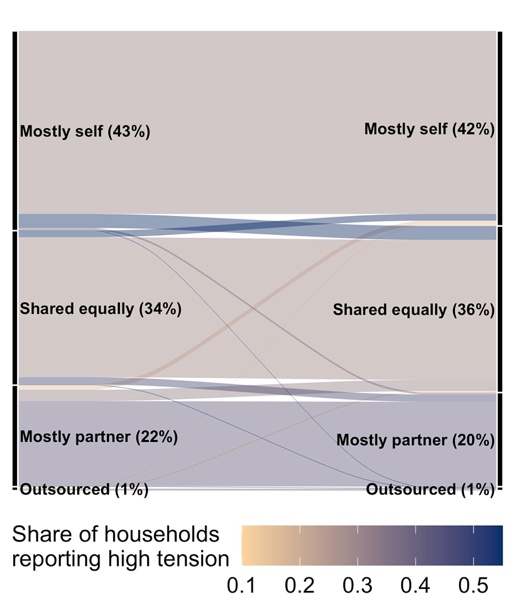
**Supplementary Figure S6: Changes in division of laundry from before to during the lockdown, coloured by share of household reporting high tension**

**ITA**

**UK**

**US**

*Notes:* The above Sankey diagrams report changes in laundry allocation from before the lockdown (left-hand side of each diagram) to during the lockdown (right-hand side of each diagram) for each of the countries surveyed. Diagram flows are coloured by the share of respondents reporting high household tensions specifically related to the allocations of household tasks. Darker lines correspond to subsets with higher reported household tensions, and are useful in capturing the effect of task reallocation in lockdown. Source: online survey in Italy, UK, USA. N = 2,524.

1. University of Zürich [↑](#footnote-ref-1)
2. University of Reading [↑](#footnote-ref-2)
3. University of Birmingham [↑](#footnote-ref-3)
